# Electroacupuncture alleviates functional constipation by upregulating host-derived miR-205-5p to modulate gut microbiota and tryptophan metabolism

**DOI:** 10.3389/fmicb.2025.1517018

**Published:** 2025-02-05

**Authors:** Lu Wang, Menghan Xi, Wei Cao, Haiyan Qin, Di Qin, Shuai Chen, Siyuan Zhou, Yujun Hou, Ying Chen, Xianjun Xiao, Qianhua Zheng, Dehua Li, Ying Li

**Affiliations:** ^1^Department of Acupuncture, Hospital of Chengdu University of Traditional Chinese Medicine, Chengdu, Sichuan, China; ^2^Acupuncture and Tuina School, Chengdu University of Traditional Chinese Medicine, Chengdu, Sichuan, China; ^3^Department of Acupuncture and Moxibustion, Chengdu Pidu District Hospital of TCM/The Third Clinical Medical College of Chengdu University of TCM, Chengdu, Sichuan, China; ^4^Center of Preventive Medicine, Chengdu Integrated TCM and Western Medicine Hospital, Chengdu, Sichuan, China; ^5^School of Health Preservation and Rehabilitation, Chengdu University of Traditional Chinese Medicine, Chengdu, Sichuan, China

**Keywords:** electroacupuncture, functional constipation, miR-205-5p, *Lactobacillus reuteri*, tryptophan metabolism

## Abstract

Electroacupuncture (EA) has shown promise as a treatment for Functional constipation (FC), with growing evidence suggesting it may enhance gut motility. MicroRNAs (miRNAs) serve as key regulatory molecules mediating host-microbiota interactions. However, the specific fecal miRNAs regulating microbiota composition and metabolism in EA-treated constipated mice, along with their key targets, remain unidentified. We examined fecal microbiome composition, metabolism, and colonic miRNA expression in loperamide-induced constipated mice and EA-treated mice to identify differentially expressed miRNAs and assess their relationships with microbial abundance, metabolism, and gut motility. An antibiotic cocktail and adeno-associated virus were employed to interfere with the gut microbiota and target miRNA in vivo, thereby validating the proposed mechanism. Our results indicate that miR-205-5p, significantly upregulated in fecal and colonic tissues of EA-treated constipated mice, promotes intestinal motility in a microbiome-dependent manner. Specifically, EA promoted the growth of *Lactobacillus reuteri*, enriched in the feces of constipation-recovered mice, through host-derived miR-205-5p regulation. Furthermore, *Lactobacillus reuteri* and its tryptophan metabolites (indole-3-acetamide, indole-3-acetic acid, and indole-3-carboxaldehyde) alleviated loperamide-induced constipation. These findings underscore the pivotal role of host-derived miR-205-5p in modulating microbial composition and tryptophan metabolites to enhance intestinal motility through EA.

## 1 Introduction

Functional constipation (FC) is a non-organic digestive disorder that significantly impairs quality of life. Clinical symptoms are primarily characterized by infrequent bowel movements, difficult or painful defecation, and/or dry, hard stools. With advancements in diagnostic criteria and an aging population, the prevalence of FC continues to rise (Chen et al., 2022; Palsson et al., 2020). Clinically, current treatments for FC include dietary fiber supplementation, adequate hydration, lifestyle modifications, laxatives, stimulants, and, in some cases, surgical interventions. However, these treatments frequently result in side effects, variable efficacy, and significant individual differences (Chang et al., 2023; Vriesman et al., 2020). Managing FC remains challenging due to its complex pathogenesis.

The human gastrointestinal tract contains trillions of microbes that interact intricately with the host. This microbiota constitutes a highly dynamic ecosystem that is crucial for regulating physiological processes such as immune function, nutrient absorption, and intestinal motility (Waclawikova et al., 2022; Wang D. et al., 2024; Wang H. et al., 2024; Wang J. et al., 2024; Xue et al., 2024). Studies have demonstrated that gut microbiota disturbances are strongly associated with constipation and that changes specific flora, such as *Bacteroides thetaiotaomicron* and *Lactobacillus rhamnosus GG*, may influence intestinal peristalsis and alleviate constipation symptoms (Aktar et al., 2020; Rice et al., 2022). Conversely, host biological factors (e.g., miRNA, immune molecules, and mucous molecules) can regulate gut microbiota composition and function, thus impacting gut dynamics and overall health (Zhang C. et al., 2023; Zhang X. et al., 2023). Elucidating the mechanisms by which these host factors modulate gut microbiota and contribute to disease development is crucial for advancing preventive and therapeutic strategies.

MicroRNAs (miRNAs) are small, non-coding RNAs that regulate numerous biological processes post-transcriptionally, including immune responses, inflammation, and cellular homeostasis. Notably, miRNAs are involved in modulating the gut microbiota, a critical factor in maintaining intestinal health (Aguilar et al., 2019; Santos et al., 2020). The interaction between host-derived miRNAs and gut microbiota constitutes a complex and dynamic relationship. miRNAs specifically regulate bacterial gene expression by destabilizing mRNA and inhibiting post-transcriptional regulatory proteins, thereby affecting bacterial growth (Liu et al., 2016). Conversely, bacterial pathogens manipulate host miRNA expression to promote their survival, replication, and persistence (Aguilar et al., 2019). This highlights the crucial role of miRNAs in regulating gut microbiota through host–microbe interactions, which influence gastrointestinal health in ways yet to be fully understood. Preliminary clinical studies suggested that specific host miRNAs, such as miR-205-5p, miR-493-5p, miR-215-5p, miR-184, and miR-378c, influence constipation development by modulating the gut microbiota (Yao et al., 2023). Further investigation into how host miRNAs influence constipation may offer valuable insights for novel therapeutic strategies.

Electroacupuncture (EA) is recognized as a promising alternative treatment for FC with studies demonstrating that its therapeutic effects can last up to 24 weeks after an 8-week treatment period (Liu et al., 2021). However, as with other diseases where acupuncture has proven effective, its clinical application for FC is limited by the unclear mechanism of action associated with the treatment. We observed that EA alleviates constipation symptoms in FC mice by restoring gut microbiota balance, increasing butyric acid levels, and enhancing colonic peristalsis and secretion (Xu et al., 2023). Unlike dietary interventions that directly influence gut microbes, the upstream mechanisms by which acupuncture, as a non-pharmacological treatment, exerts its effects remain unclear (Gao et al., 2023; Ross et al., 2024). Recent evidence suggests that acupuncture may exert its effects via molecular signaling pathways in the host, including the regulation of miRNA expression (Lu et al., 2024; Yu et al., 2024). Therefore, this study hypothesizes that acupuncture improves intestinal motility in constipation by modulating host miRNA expression, which subsequently regulates gut flora composition and metabolism. Using multi-omics analysis and functional validation experiments, we sought to identify critical pathways that mediate the therapeutic effects of EA. An antibiotic cocktail (Abx) and adeno-associated virus (AAV) were employed to interfere with the gut microbiota and target miRNA *in vivo*, thereby validating the proposed mechanism.

## 2 Materials and methods

### 2.1 Animals and groups

**Figure 1:**
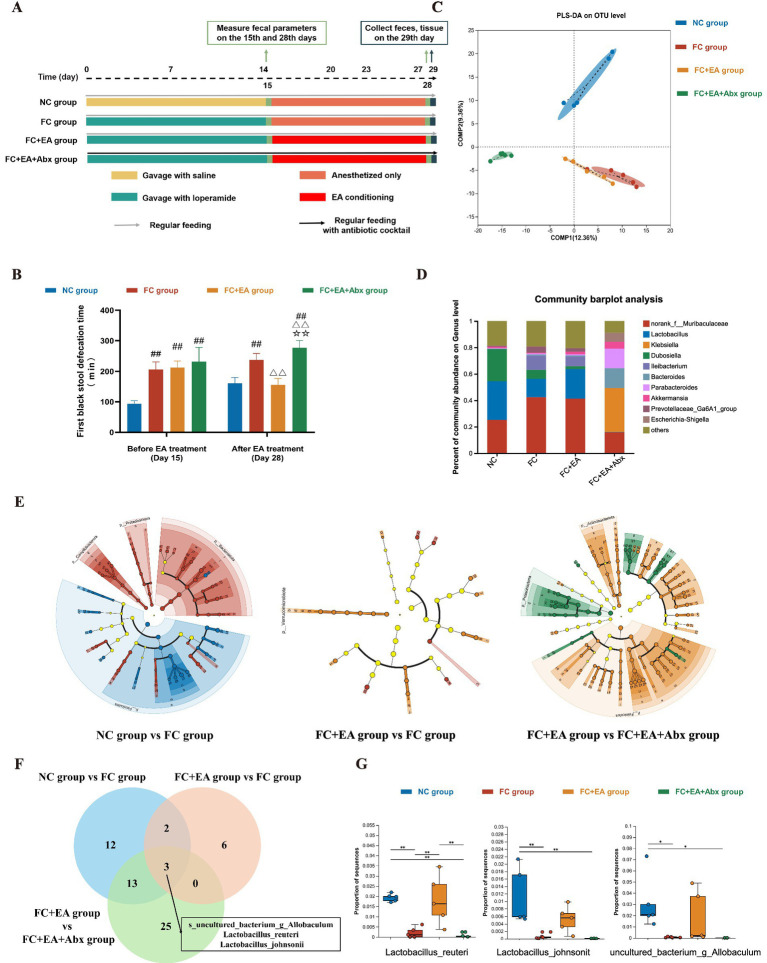
EA promotes gut motility and alleviates loperamide-induced constipation by regulating fecal microbiota composition. **(A)** Schematic design of experiment 1. **(B)** The first black stool defecation time. Data were analyzed with one-way ANOVA (*n* = 8) vs. NC group: ^##^*p* < 0.01; vs. FC group: ^△△^*p* < 0.01; vs. FC + EA group: ^☆☆^*p* < 0.01. **(C)** Partial Least Squares Discriminant Analysis (PLS-DA) based on OTUs. **(D)** Relative abundance of bacteria classified at a gunes-level taxonomy. **(E)** Linear Discriminant Analysis Effect Size (LEfSe) analysis between groups. **(F)** Veen plots for differential species in the three sets of comparisons. **(G)** Relative expression of *Lactobacillus_reuteri*, *Lactobacillus_johnsonii*, *uncultured_bacterium_g_Allobaculum*. Data were analyzed with Kruskal-Wallis rank sum test. ^*^p < 0.05; ^**^p < 0.01; ^***^p < 0.001. EA, electroacupuncture; FC, functional constipation; NC, normal control; Abx, antibiotic cocktail.

**Figure 2:**
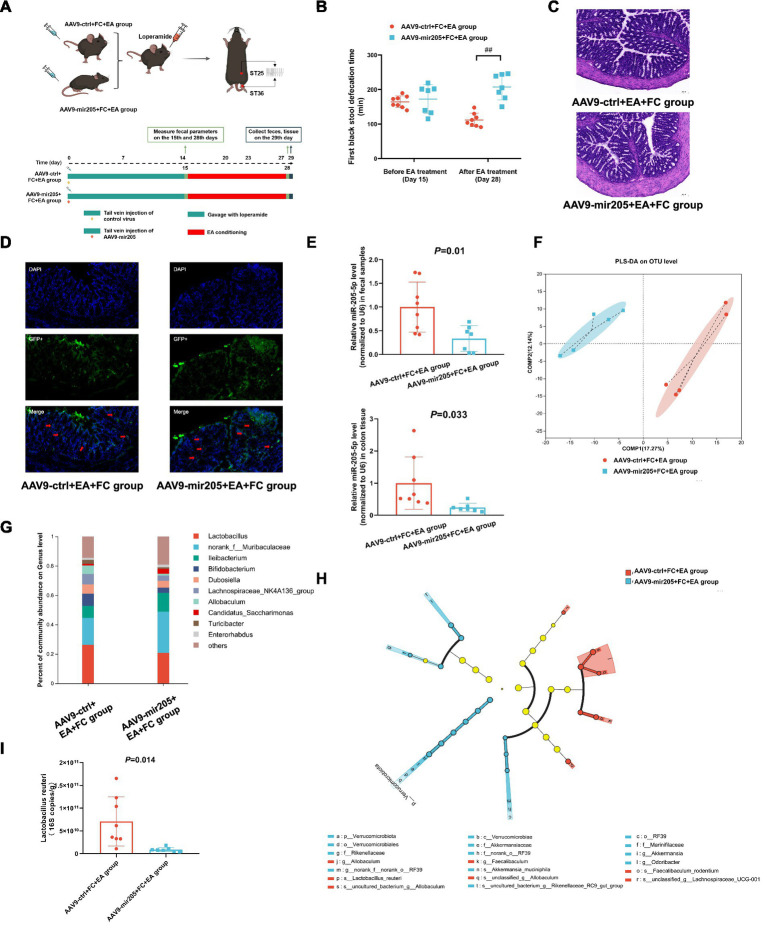
Knockdown colonic miR-205 inhibit the effects of EA on gut motility and *Lactobacillus reuteri* in loperamide-induced constipation mice. **(A)** Schematic design of experiment 2. **(B)** The first black stool defecation time. vs. AAV9-ctrl+FC + EA group:^##^*p* < 0.01. **(C)** Representative colonic morphology. **(D)** AAV9-mediated transduction efficacy following IP injection as visualized in frozen ring sections. The red arrows represent GFP+ cells. **(E)** Relative expression of miR-205-5p. **(F)** Partial Least Squares Discriminant Analysis (PLS-DA) based on OTUs. **(G)** Relative abundance of bacteria classified at a gunes-level taxonomy. **(H)** Linear Discriminant Analysis Effect Size (LEfSe) analysis between groups. **(I)** Absolute expression of *Lactobacillus_reuteri*. Data were analyzed with Unpaired *t*-test.

**Figure 3:**
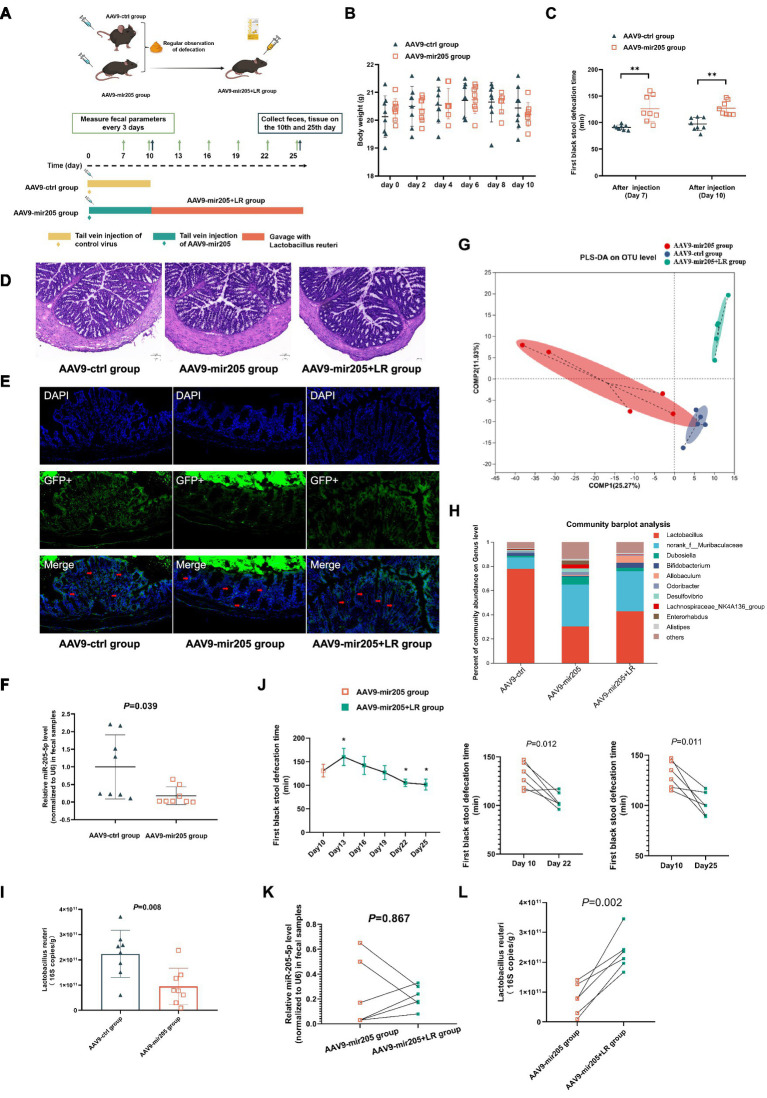
*Lactobacillus reuteri* restores the inhibitory effect of AAV9-mir205 on gut motility in normal mice. **(A)** Schematic design of experiment 3. **(B)** Body weight change. **(C)** The first black stool defecation time. vs. AAV9-ctrl group: ^**^p < 0.01. **(D)** Representative colonic morphology. **(E)** AAV9-mediated transduction efficacy following IP injection as visualized in frozen ring sections. The red arrows represent GFP+ cells. **(F)** Relative expression of miR-205-5p. **(G)** Partial Least Squares Discriminant Analysis (PLS-DA) based on OTUs. **(H)** Relative abundance of bacteria classified at a gunes-level taxonomy. **(I)** Absolute expression of *Lactobacillus reuteri*. **(J)** The first black stool defecation time after gavaging *Lactobacillus reuteri*. vs. AAV9-205 group:^*^p < 0.05. **(K)** Relative expression of miR-205-5p after gavaging *Lactobacillus reuteri*. **(L)** Absolute expression of *Lactobacillus reuteri* after gavaging *Lactobacillus reuteri.* Data were compared between the AAV9-ctrl group and the AAV9-mir205 group using an unpaired *t*-test. The AAV9-mir205 group and the AAV9-mir205 + LR group were compared using a paired *t*-test.

C57BL/6 mice (16–20 g, 6–8 weeks old, equal numbers of males and females) were purchased from Chengdu Dossy Experimental Animals Co., Ltd. (Chengdu, China) and housed under standard laboratory conditions (12-h light/dark cycle, 22 ± 2°C, free access to food and water). The experimental groups were structured as follows: Experiment 1 sought to identify the target miRNA, its downstream bacterial species, and metabolic pathways involved in EA treatment of FC using multi-omics data. Mice were divided into four groups: normal control (NC) group, FC group, FC + EA group, and FC + EA + Abx group (*n* = 8 per group). Experiment 2 investigated whether EA’s regulation of gut motility and microbiota in FC mice is mediated by host-derived miR-205-5p. Adeno-associated virus serotype 9 with knockdown miR-205 (AAV9-mir205) was administered via tail vein injection to determine its potential to antagonize the effects of EA. Mice were randomly allocated into the AAV9-ctrl+FC + EA group (*n* = 8) and AAV9-mir205 + FC + EA groups (*n* = 8). One mouse in the AAV9-mir205 + FC + EA group died within 24 h of receiving the tail vein injection of the AAV9-mir-205. The potential causes were hypothesized to include stress from the injection procedure, tail vein injury, or individual variability in dose sensitivity to AAV9. To mitigate similar occurrences, tail vein injection techniques were optimized, and postoperative health monitoring of mice was intensified in subsequent experiments. Experiment 3 examined the effects of AAV9-mir-205 on gut motility, microbiota, and metabolism in normal mice. Mice were randomly divided into the AAV9-ctrl group and AAV9-mir205 group (*n* = 8 per group). All animals were assigned to their respective groups through a computer-generated randomization sequence. Researchers conducting the experiments and analyzing data were blinded to group assignments. All experimental procedures were approved by the Animal Ethics Committee of Chengdu University of Traditional Chinese Medicine (2021-59) and conformed to the guidelines of the National Institutes of Health (NIH) for the care and use of laboratory animals. The detailed experimental process is illustrated in Figures 1A, 2A, 3A, respectively.

### 2.2 FC and Abx mouse models

A suspension of loperamide hydrochloride (0.98 mg/mL) was administered via gavage to establish the FC model at a dose of 9.8 mg/kg body weight, twice daily for 14 days (Li et al., 2023). During the EA intervention period, the same dose was administered once daily in the morning, 30 min prior to the intervention, to maintain the model for 12 days. Preliminary experiments confirmed that once-daily administration during the 12-day intervention period was sufficient to maintain the constipation model. Mice in the FC + EA + Abx group received an antibiotic cocktail to establish a pseudo-germ-free model. Neomycin sulfate, ampicillin, metronidazole and vancomycin hydrochloride were mixed at concentrations of 1, 1, 1, and 0.5 g/L, respectively, with the solution replaced twice a week. On the 15th day, fecal parameters were measured and compared to those of the NC group to determine whether the FC model was successfully established.

### 2.3 Electroacupuncture treatment

EA treatment was conducted as previously described (Wang L. et al., 2023; Wang M. et al., 2023). Acupoints Tianshu (ST 25) and Shangjuxu (ST 37) were selected for this study. The mice were anesthetized with isoflurane gas before treatment. Stainless steel acupuncture needles (0.25 mm in diameter, 13 mm in length, Hwato, Suzhou, China) were inserted unilaterally into specific acupoints at a depth of 4–5 mm. The needles were then connected to an EA device (SDZ-V, Hwato, Suzhou, China) using alligator clips. The EA stimulation parameters were set as following: 1 mA, 3/15 Hz alternating frequency, 30 min duration. Mice in the EA treatment groups received stimulation once daily for 2 weeks, with a 2-day rest period after every 5 treatments. EA treatment was conducted at a consistent time each morning (7 a.m. to 9 a.m.), while the other two groups underwent anesthesia only during the same time frame.

### 2.4 Adeno-associated virus serotype 9 injection

Recombinant AAV9 vectors encoding miR-205-5p or the control were sourced from Saiye Biotechnology Co., Ltd. The viral titer was specified as 2E+13 GC/mL.

The viral suspension was diluted in sterile saline to a final concentration of 5E+12 GC/mL prior to injection. After 7 days of acclimatization feeding, mice were administered approximately 0.2 mL of AAV9 suspension via the tail vein injection under isoflurane gas anesthesia. Following injection, the mice were monitored until full recovery from anesthesia and were then returned to their home cages.

### 2.5 *Lactobacillus reuteri* gavage

Each mouse was administered 200 μL of the *Lactobacillus reuteri* DSM 17938 drops (#22DB336, BioGaia, Sweden), equivalent to 1 × 10^8^ CFU of *Lactobacillus reuteri*, once daily. A sterile, flexible gavage needle (20-gage, 1.5-inch length) was used for gavage, carefully inserted into the esophagus to deliver the bacterial suspension directly into the stomach.

### 2.6 Measurement of the first black stool defecation time

Following a 12-h fasting period, each mouse was administered 0.1 mL of activated charcoal suspension via gavage. The mice were subsequently placed in individual cages, and the time of first black stool defecation was recorded (Wang L. et al., 2023; Wang M. et al., 2023).

### 2.7 16S rRNA sequencing

Genomic DNA was extracted from fecal samples, with its concentration and purity assessed using electrophoresis and a NanoDrop 2000 spectrophotometer. Library construction and sequencing were outsourced to Majorbio Bio-Pharm Technology Co., Ltd. (Shanghai, China). Full-length PCR amplification of the 16S rRNA gene was conducted using 338F (5′-ACTCCTACGGGAGGCAGCAG-3′) and 806R (5′-GGACTACHVGGGTWTCTAAT-3′) primers, followed by library construction and sequencing. All sequencing data were uploaded to the Majorbio Cloud Platform for further analysis (Ren et al., 2022). Sequencing results were clustered into operational taxonomic units (OTUs) at 97% sequence similarity using UPARSE 7.1, with chimeras removed during the process. Taxonomic annotation of OTUs was performed using the RDP classifier with the Silva 16S rRNA gene database at a confidence threshold of 70%. Partial Least Squares Discriminant Analysis (PLS-DA) based on OTUs was employed to evaluate microbial community structures similarity between samples. Community composition differences between groups were analyzed at the genus level based on species abundance means within each group. Differential species analysis was performed using Linear Discriminant Analysis Effect Size (LEfSe) analysis to identify significant differences in abundance across phylum to species level, with thresholds of LDA > 3 and *p* < 0.05. The Kruskal-Wallis rank sum test, with FDR correction, was applied to evaluate differences between multiple groups.

*Post hoc* multiple comparisons were conducted using “Tukeykramer,” with significance set at *p* < 0.05.

### 2.8 MiRNAs extraction and sequencing

Total RNA extraction, quantitative determination and quality control, library construction and sequencing were performed by Kangcheng Biological Co., Ltd. (Shanghai, China). Subsequently, miRDeep2 software was utilized to align sequencing results to the miRBase v22 database and calculate read counts. Differentially expressed miRNAs between groups were analyzed using DESeq2 package in the R software, with thresholds set at adjust *p*-value ≤ 0.05 and |Log2FC| ≥ 0.5.

### 2.9 Quantitative real-time PCR

Total RNA was extracted from fecal and colonic samples for miRNA analysis using RNAiso Plus (#9109, Takara). RNA was reverse-transcribed into miRNA cDNA synthesis using All-in-One miRNA First-Strand cDNA Synthesis Kit 2.0 (#QP114, GeneCopoeia, China). Quantification of miRNA cDNAs was conducted via real-time PCR using Hieff^®^ qPCR SYBR Green Master Mix (Low Rox Plus) (#11202ES08, GeneCopoeia, China) on ViiA 7 Real-Time PCR System (ABI, United States), following the manufacturer’s protocol. Bacterial DNA for gut bacteria analysis was extracted from feces using the E.Z.N.A.^®^ Soil DNA Kit (Omega Bio-Tek, United States) with quantity and quality assessed using a NanoDrop 2000 spectrophotometer (Thermo Fisher Scientific). Quantitative PCR (qPCR) was conducted on an ABI7300 fluorescence quantitative PCR system (Applied Biosystems, United States) using ChamQ SYBR Color qPCR Master Mix (2X) (Novozymes, China). The specific Primer sequences were listed in Supplementary Table S1.

### 2.10 Metabolomic analysis

Metabolites extraction from feces, untargeted liquid chromatography–tandem mass spectrometry (LC–MS/MS) analysis, and data preprocessing and annotation were outsourced to Majorbio Bio-Pharm Technology Co., Ltd. (Shanghai, China). All data were uploaded to the Majorbio Cloud Platform for subsequent analysis (Ren et al., 2022). Metabolic profiling was conducted using orthogonal partial least squares discriminant analysis (OPLS-DA) for anions and cations, with Paletto conversion applied for data transformation. The model was validated using a 200-cycle permutation test. Differential metabolite screening identified significant metabolites between groups (NC group vs. FC group, FC group vs. FC + EA group, FC + EA group vs. FC + EA + Abx group), based on variable importance projection (VIP) >1 from OPLS-DA analysis, *p*-value < 0.05, and fold change (FC) >1 from Student’s t-test. Pathway enrichment analysis was conducted by mapping common differential metabolites to the KEGG database (Kyoto Encyclopedia of Genes and Genomes, http://www.genome.jp/kegg/). Multiple testing correction was performed using the “Bonferroni” method, and “Relative Betweenness Centrality” was applied as the topological method. Likewise, targeted metabolomics analysis of fecal samples was outsourced to Shanghai Majorbio Bio-pharm Technology Co., Ltd. (Shanghai, China). Fecal metabolites in tryptophan pathway were characterized using UHPLC–MS/MS, as detailed in previous publications (Cheng et al., 2022; Liu et al., 2024a).

### 2.11 Intestinal morphology

The colon tissue samples were dehydrated and fixed prior to embedding in OCT compound. Tissue sections were prepared using a cryostat (Thermo Fisher, United States), and stained with hematoxylin and eosin (H&E) to observe morphological changes.

### 2.12 Immunofluorescence staining for AAV9 detection

Sections were washed with PBS (pH 7.4) and incubated with DAPI. GFP expression in the mucosa and submucosa was visualized using a panoramic pathology scanner (PanoVIEW VS200, Olympus, Japan) (Ma et al., 2022).

### 2.13 Statistical analysis

Unless otherwise specified in the methods sections, all statistical analyzes were conducted using Prism 8 (GraphPad Software, San Diego, CA). Multiple group comparisons were conducted using least significant difference (LSD) and Kruskal-Wallis rank-sum tests based on the results of normality and homogeneity of variance tests. Similarly, two-group comparisons were analyzed using unpaired *t*-tests, Welch’s corrected unpaired *t*-tests, or Mann–Whitney non-parametric tests, except for the comparison between the AAV9-mir205 group and the AAV9-mir205 + LR group, which was performed using a paired t-test. A *p*-value of < 0.05 was considered statistically significant.

## 3 Results

### 3.1 EA promotes gut motility and alleviates loperamide-induced constipation by regulating fecal microbiota composition

To evaluate the therapeutic effect of EA treatment on loperamide-induced constipation in mice, gut motility was assessed using the defecation time for the first black stool, 6-h fecal pellet count, wet weight. After a 14-day loperamide intervention, the FC group exhibited a significantly longer defecation time for the first black stool compared with the NC group. Similarly, the fecal pellet count and wet weight over 6 h significantly reduced in the FC group compared to the NC group (Figure 1B; Supplementary Figure S1). Thus, loperamide successfully induced gastrointestinal transit disorder in FC model mice. After 2 weeks of EA treatment, all gut motility indicators significantly improved in the FC + EA group, compared to the FC group (Figure 1B; Supplementary Figure S1). In addition, the FC + EA + Abx group mice, treated with a broad-spectrum antibiotic in drinking water, exhibited a significantly longer defecation time for the first black stool and reduced 6-h fecal pellet count and wet weight compared to the FC + EA group (Figure 1B; Supplementary Figure S1). These findings suggest that gut microbiota mediates the positive effects of EA on gut motility, consistent with previous reports (Xu et al., 2023).

Intestinal microbial alterations were investigated using 16S rDNA sequencing of feces collected from each group of mice. Alpha diversity analysis revealed significant differences between the FC + EA + Abx group and all three other groups (Supplementary Figure S2A). Similarly, microbial beta-diversity analysis showed that the microbial structure in the FC + EA + Abx group was distinct from the other groups, indicating that antibiotics dramatically affected gut microbiota composition (Figure 1C). PLS-DA also demonstrated that the NC group was clearly distinguishable from the FC and FC + EA groups, clustering into different taxa (Figure 1C). Despite some overlap in microbial structure between the FC and FC + EA groups, significant differences were observed in the relative abundance of specific intestinal microbes. To identify the specific intestinal microbes that were influenced by EA treatment, we compared the relative abundance of gut microbial species identified in the feces of each group of mice. At the genus level, the FC + EA + Abx group was dominated by *Klebsiella* spp., *norank_f__Muribaculaceae* spp., and *Bacteroides* spp., whereas the other groups were dominated by *norank_f__Muribaculaceae* spp., *Lactobacillus* spp., and *Dubosiella* spp. (Figure 1D). Notably, loperamide modeling reduced *Lactobacillus* abundance in mouse feces, which was restored following EA treatment (Figure 1D). Additionally, broad-spectrum antibiotics negated the positive effects of EA on *Lactobacillus*. LEfSe was employed to identify differential species between the NC group and FC group, FC group and FC + EA group, and FC + EA group and FC + EA + Abx group (Figure 1E; Supplementary Figure S3). The intersection of all species-level differences from the three comparisons was analyzed. As depicted in the Venn diagrams (Figure 1F), three differential species were identified: *s_uncultured_bacterium_g_Allobaculum*, *s_Lactobacillus_reuteri*, *s_Lactobacillus_johnsonii*. The relative abundance of these three differential species were significantly reduced in the FC group compared to NC group. However, only the abundance of *Lactobacillus reuteri* was restored following EA treatment (Figure 1G). Compared to the FC + EA group, the relative abundance of *Lactobacillus reuteri* was significantly lower in the FC + EA + Abx group, suggesting that EA’s regulation of *Lactobacillus reuteri* was inhibited by antibiotics. Previous studies have shown that administrating *Lactobacillus reuteri* DSM 17938 alleviates clinical symptoms in patients with constipation (Riezzo et al., 2019).

### 3.2 Identification of differentially expressed colonic miRNA in loperamide-induced mice regulated by EA

**Figure 4:**
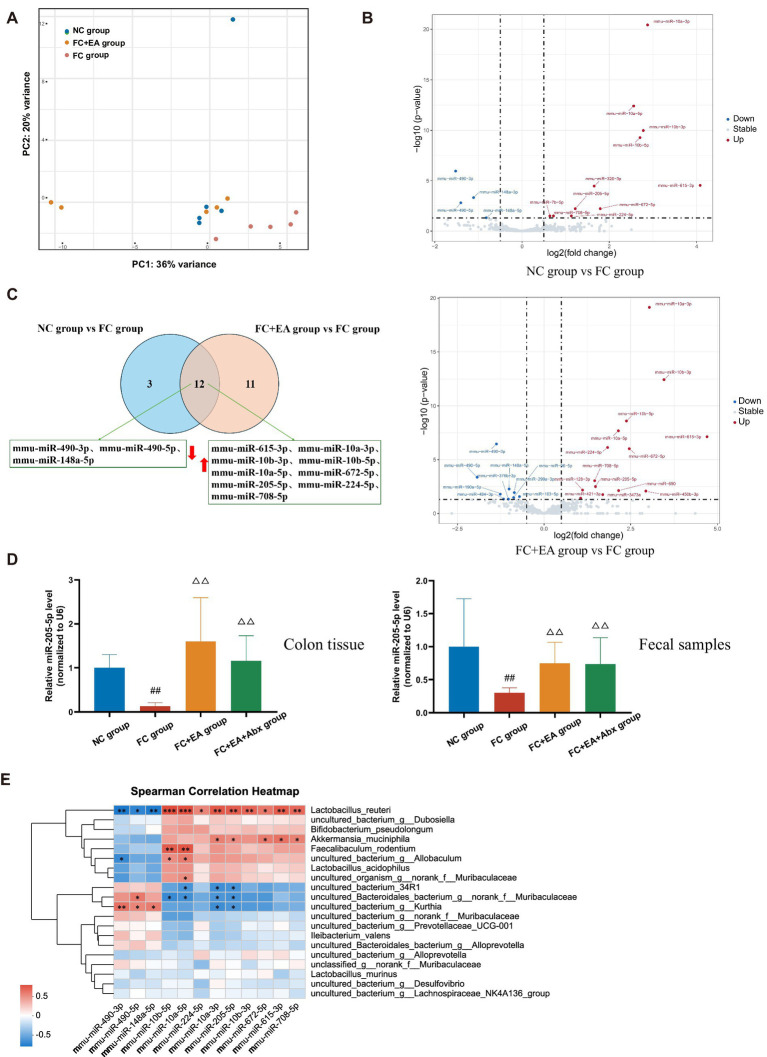
Identification of differentially expressed colonic miRNA in loperamide-induced mice regulated by EA. **(A)** Principal coordinates analysis (PCoA) based on weighted UniFrac distance. **(B)** Volcano plot of colonic miRNAs among the groups. **(C)** Veen plots for differential miRNAs in colonic tissues on FC group when compared to those of either NC group or FC + EA group. **(D)** Relative expression of miR-205-5p. Data were analyzed with one-way ANOVA (*n* = 8) vs. NC group: ^##^*p* < 0.01; vs. FC group: ^△△^*p* < 0.01. **(E)** Spearman correlation heatmap between differential species and differential miRNAs.

Previous studies have demonstrated altered fecal miRNA profiles in FC patients compared to healthy individuals (Yao et al., 2023). Host intestinal epithelial cells and homeodomain-only protein homeobox (HOPX)-positive cells are primary sources of miRNAs that influence the growth of intestinal luminal microbiota (Zhang C. et al., 2023; Zhang X. et al., 2023). To identify host-derived miRNAs regulated by EA in alleviating loperamide-induced constipation, small RNA sequencing was performed on colonic tissues from mice in the NC, FC, and FC + EA groups. PCA revealed that the FC group formed distinct clusters from the FC + EA and NC groups, while the NC and FC + EA groups overlapped (Figure 4A). The analysis showed that 11 miRNAs were upregulated and 4 were downregulated in the NC group compared to the FC group, while 14 miRNAs were upregulated and 9 were downregulated in the FC + EA group compared to the FC group (Figure 4B; Supplementary Tables S2, S3). Among these changes, 9 miRNAs (mmu-miR-615-3p, mmu-miR-10a-3p, mmu-miR-10b-3p, mmu-miR-10b-5p, mmu-miR-10a-5p, mmu-miR-672-5p, mmu-miR-205-5p, mmu-miR-224-5p, and mmu-miR-708-5p) were upregulated, while 3 miRNAs (mmu-miR-490-3p, mmu-miR-490-5p, and mmu-miR-148a-5p) were downregulated in the FC group compared to the NC or FC + EA groups (Figure 4C). Combined with prior sequencing of fecal miRNAs from FC patients, miR-205-5p appears to be a key miRNA in EA treatment (Yao et al., 2023). Increased miR-205-5p levels in colon tissue and feces of the FC + EA group compared to the FC group were confirmed by qPCR (Figure 4D). Antibiotic treatment significantly elevated miR-205-5p expression levels in colon tissues and feces of the FC + EA + Abx group compared to the FC group (Figure 4D). This suggests that removal of the intestinal flora does not alter EA’s effect on miR-205-5p expression in the intestinal lumen.

To investigate the relationship between gut microbiota and colonic miRNAs, we correlated the 12 differentially expressed miRNAs with the top 20 gut microbial species ranked by total abundance. As shown in Figure 4E, the three downregulated miRNAs were significantly negatively correlated with *Lactobacillus reuteri* abundance, whereas the nine upregulated miRNAs, including miR-205-5p were significantly positively correlated with *Lactobacillus reuteri* abundance. Thus, host-derived miR-205-5p may influence the growth of *Lactobacillus reuteri*, contributing to the regulation of intestinal function.

### 3.3 Knockdown colonic miR-205 inhibit the effects of EA on gut motility and *Lactobacillus reuteri* in loperamide-induced constipation mice

To determine whether miR-205-5p influences the therapeutic effects of EA in loperamide-induced constipation, AAV9-mir205 was synthesized and administered via tail vein injection (Figure 2A). The results showed a significant prolongation of the first black stool defecation time in the AAV9-mir205 + EA + FC group (Figure 2B). Histopathological examination revealed no significant pathological damage to colonic tissues in either group, suggesting that AAV9 exhibited minimal toxicity (Figure 2C). Immunofluorescence analysis indicated that GFP^+^ cells were present in the colonic tissues of both groups, primarily in the mucosal and submucosal layers, with sparse distribution in the muscularis propria (Figure 2D). qPCR analysis revealed significantly reduced miR-205-5p expression in colon tissue and fecal samples of AAV9-mir205 + FC + EA group compared to the AAV9-ctrl+FC + EA group (Figure 2E). Tail vein injection of AAV9 successfully targeted the colonic tissues of mice and was functionally effective. 16S rDNA sequencing was performed to analyze overall microbial composition. While alpha diversity showed no significant differences (Supplementary Figure S4), PLS-DA analysis of beta diversity revealed that the fecal microbial structure in the AAV9-mir205 + FC + EA group was distinct from that in the AAV9-ctrl+FC + EA group (Figure 2F). At the genus level, intestinal flora in both groups was dominated by *Lactobacillus* spp., *norank_f__Muribaculaceae* spp., and *Ileibacterium* spp. The relative abundance of *Lactobacillus* spp. was significantly lower in the AAV9-mir205 + FC + EA group (20.7%) compared to the AAV9-ctrl+FC + EA group (26.2%) (Figure 2G). LEfSe analysis showed significant enrichment of several strains, including *Lactobacillus reuteri*, in fecal samples from the AAV9-mir205 + FC + EA group compared to the AAV9-ctrl+FC + EA group (Figure 2H). qPCR results indicated a significant reduction in the absolute expression of *Lactobacillus reuteri* in feces from AAV9-mir205 + FC + EA group (Figure 2I). Thus, the AAV9-mir205 inhibited the effects of EA on gut motility and *Lactobacillus reuteri* in mice with loperamide-induced constipation.

### 3.4 *Lactobacillus reuteri* restores the inhibitory effect of AAV9-mir205 on gut motility in normal mice

No prior studies have investigated the role of miR-205-5p in the regulation of intestinal motility and *Lactobacillus reuteri*. AAV9-mir205 was first administered to untreated mice by tail vein injection, showing minimal effects on their body weight (Figure 3B). Since the onset of AAV9 typically occurs at 7 days post-injection, intestinal motility was assessed from day 7 onwards. The defecation time for the first black stool was significantly prolonged on days 7 and 10 post-injection in AAV9-mir205 mice compared to controls (Figure 3C). Results of HE staining and immunofluorescence staining were consistent with previous findings, indicating that AAV9 successfully targeted the mucosal epithelium of colon tissue without disrupting the tissue structure (Figures 3D,E). On day 10 post-administration, miR-205-5p levels in feces were lower in AAV9-mir205 mice than in controls, confirming its role in intestinal motility regulation (Figure 3F). To investigate the role of miR-205-5p in shaping the intestinal microbiota, alpha diversity showed no significant differences, whereas beta diversity revealed some separation between the groups (Supplementary Figure S5; Figure 3G). At the genus level, both AAV9-ctrl and AAV9-mir205 groups were dominated by *Lactobacillus* spp. and *norank_f__Muribaculaceae* spp., with relative abundances of 77.8% vs. 30.2% and 9.5% vs. 34.6%, respectively (Figure 3H). LEfSe analysis identified significant differences at multiple species levels, with the abundance of species, such as *Lactobacillus reuteri*, *Lactobacillus intestinalis*, and *Lactobacillus_johnsonii*, significantly reduced in the miRNA-205 group compared to controls (Supplementary Figure S6). qPCR results confirmed a significant reduction in the absolute expression of *Lactobacillus reuteri* in feces from AAV9-mir205 mice (Figure 3I). Diminished gut motility in miR-205 knockdown mice was associated with reduced *Lactobacillus reuteri* expression in the intestine.

To confirm the role of *Lactobacillus reuteri* in miR-205-5p-mediated regulation of intestinal motility, AAV9-mir205 group mice were gavaged with *Lactobacillus reuteri* DSM17938 (BioGaia), forming the AAV9-mir205 + LR group. As shown in the Figure 3J, pairwise comparisons indicated that the defecation time for the first black stool was significantly reduced on day 12 and day 15 of consecutive *Lactobacillus reuteri* gavage compared to pre-gavage. Immunofluorescence results confirmed persistent colonization of colonic tissues by AAV9-mir205 (Figure 3E). Pairwise comparisons with the AAV9-mir205 group revealed no statistically significant difference in fecal miR-205-5p expression levels in the AAV9-mir205 + LR group (*p* > 0.05, Figure 3K). These results suggest that the miR-205 knockdown effect of AAV9 was sustained. Gavage of *Lactobacillus reuteri* significantly altered the microbial structure, as indicated by 16S rDNA sequencing of feces (Supplementary Figure S5). Species difference analysis showed increased abundance of *Lactobacillus reuteri*, *Akkermansia muciniphila*, and *Bifidobacterium animalis* in the feces of the AAV9-mir205 + LR group compared to the AAV9-mir205 group (Supplementary Figure S7). As seen in the Figure 3L, qPCR further confirmed increased *Lactobacillus reuteri* abundance in feces. These findings demonstrate that *Lactobacillus reuteri* successfully colonized the intestine and counteracted the inhibitory effect of AAV9-mir205 on gut motility.

### 3.5 Screening of *Lactobacillus reuteri* related fecal metabolites in loperamide-induced constipation mice regulated by EA

**Figure 5:**
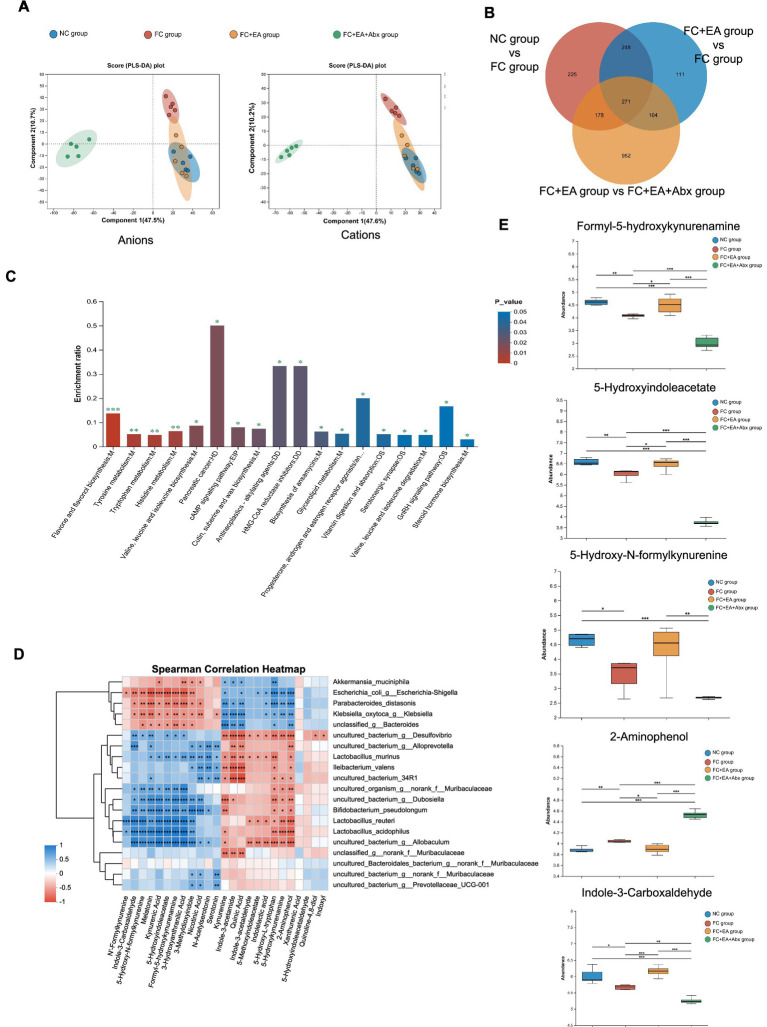
Screening of *Lactobacillus reuteri* related fecal metabolites in loperamide -induced constipation mice regulated by EA. **(A)** Score graph of the PLS-DA analysis model for the four groups. **(B)** Veen plots for differential species in the three sets of comparisons. **(C)** KEGG enrichment analysis plot of differential metabolites. **(D)** Spearman correlation heatmap between differential species and differential tryptophan metabolites. **(E)** Relative abundance of representative differential tryptophan metabolites. ^*^p < 0.05, ^**^p < 0.01, ^***^p < 0.001.

Previous studies suggest that gut bacteria regulate intestinal motility through their metabolites (Waclawikova et al., 2022). To identify flora-regulation-related fecal metabolites affected by EA in relieving constipation, we conducted LC–MS untargeted metabolomics analysis on feces from loperamide-induced constipated mice with or without broad-spectrum antibiotic treatment post-EA. After preprocessing, differences in fecal metabolic profiles across the four groups were analyzed using two methods, PLS-DA and OPLS-DA, respectively. As shown in the Figure 5A, the PLS-DA model for anions and cations revealed overlapping fecal metabolic profiles between the NC group and FC + EA group, which were distinct from FC group. In contrast, the FC + EA + Abx group displayed clearly distinct fecal metabolic profiles compared to the other three groups. OPLS-DA analysis identified significant differences in fecal metabolic profiles between groups (NC group vs. FC group, FC group vs. FC + EA group and FC + EA group vs. FC + EA + Abx group, Supplementary Figure S8). Further screening identified 271 shared differential fecal metabolites between groups, as shown in Figure 5B. These metabolites were primarily associated with flavone and flavonol biosynthesis, tyrosine metabolism, tryptophan metabolism, histidine metabolism (Figure 5C). Notably, both *in vivo* and *in vitro* experiments have demonstrated that *Lactobacillus reuteri* metabolizes and activates tryptophan (Cervantes-Barragan et al., 2017; Montgomery et al., 2022). Association analysis from this study showed that *Lactobacillus reuteri*, one of the top 20 species by total abundance, was significantly linked to several tryptophan metabolites (Figure 5D).

One-way ANOVA followed by Tukey–Kramer tests identified differential tryptophan metabolites (*p* < 0.05) across the four groups (Figure 5E). Compared to the NC group, the FC group exhibited reduced levels of 4 tryptophan metabolites (Formyl-5-hydroxykynurenamine, 5-Hydroxy-N-formylkynurenine, 5-Hydroxyindoleacetate, Indole-3-Carboxaldehyde) in the feces, while 2-Aminophenol levels increased. Further comparison revealed that EA treatment reduced 2-Aminophenol levels while increasing Formyl-5-hydroxykynurenamine, 5-Hydroxyindoleacetate, and Indole-3-Carboxaldehyde levels in feces of FC model mice. Additionally, antibiotic treatment inhibited EA’s regulatory effects on these 4 tryptophan metabolites in the feces of FC mice. These findings suggest that EA’s regulation of tryptophan metabolites in the feces of constipated mice is dependent on gut microbiota.

### 3.6 *Lactobacillus reuteri* mediates the regulatory effects of EA on miR-205-5p and tryptophan metabolites in FC mice

**Figure 6:**
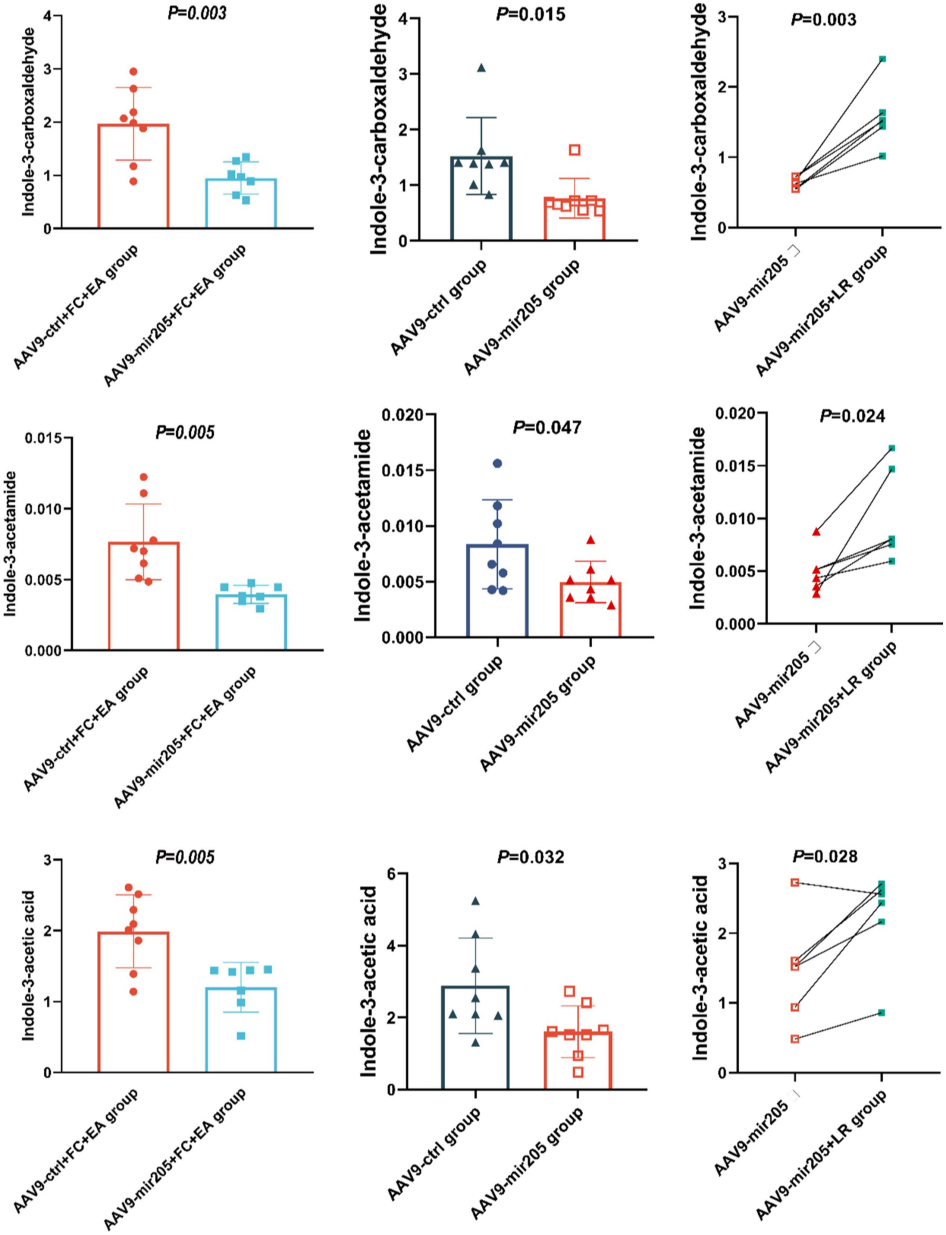
*Lactobacillus reuteri* mediates the regulatory effects of EA on miR-205-5p and tryptophan metabolites in FC mice. Absolute quantitative results of differential tryptophan metabolites between groups. Comparison of data between the AAV9-ctrl group and the AAV9-mir205 group or the AAV9-ctrl+FC + EA group and the AAV9-mir205 + FC + EA group was performed using an unpaired *t*-test. The AAV9-mir205 group and the AAV9-mir205 + LR group were compared using a paired *t*-test.

We investigated the impact of miR-205 knockdown on the EA-regulated tryptophan metabolites in the feces of loperamide-induced constipated mice. Compared to the AAV9-ctrl+FC + EA group, AAV9-mir205 + FC + EA group showed significantly reduced levels of picolinic acid (PCL 016), N-(3-indolylacetyl)-L-alanine, rac-kynurenine, kynurenic Acid, indole-3-carboxaldehyde, indole-3-acetic acid, and indole-3-acetamide (all *p* < 0.05), while tryptamine levels were significantly elevated (*p* < 0.01; Figure 6; Supplementary Table S4). Further analysis explored whether the regulation of these metabolites was associated with *Lactobacillus reuteri*. Transfection of normal mice with AAV9-mir205 resulted in significantly reduced levels of indole-3-acetamide, indole-3-carboxylic acid, indole-3-carboxaldehyde, indole-3-acetic acid, and tryptophol in the feces (all *p* < 0.05), whereas indole levels significantly increased (*p* < 0.05; Figure 6; Supplementary Table S5). After *Lactobacillus reuteri* gavage in AAV9-mir205 mice, fecal levels of 2-aminophenol, rac-kynurenine, xanthurenic acid, kynurenic acid, and 5-hydroxyindole-3-acetic acid were significantly reduced (all *p* < 0.05), while L-tryptophan, indole-3-acetamide, indole-3-acetic acid, and indole-3-carboxaldehyde levels significantly increased (all *p* < 0.05; Figure 6; Supplementary Table S6). Notably, *Lactobacillus reuteri* restored the levels of indole-3-acetamide, indole-3-acetic acid, and indole-3-carboxaldehyde reduced by AAV9-mir205 infection. Thus, the improvement of gut motility in loperamide-induced constipated mice by EA treatment, through increased levels of these 3 tryptophan metabolites, may involve host-derived miR-205-5p promoting the growth of *Lactobacillus reuteri*.

## 4 Discussion

This study demonstrated that EA enhances intestinal motility in functional constipation through the upregulation of miR-205-5p, which promotes the growth of *Lactobacillus reuteri* and regulates tryptophan metabolism. These findings provide novel insights into the mechanisms underlying EA’s therapeutic effects. EA has been widely shown to enhance intestinal motility in both FC patients and animal models (Liu et al., 2021; Wang et al., 2019; Xu et al., 2023). The treatment of FC is closely linked to the composition of the intestinal microbiota (Huang et al., 2024; Pan et al., 2022). Our study revealed that EA modulated the intestinal microbial composition of loperamide-induced constipated mice, particularly by increasing the abundance of *Lactobacillus* at the genus level. Previous clinical studies have demonstrated that *Lactobacillus* abundance may predict the efficacy of acupuncture for FC (Yan et al., 2023). Another study found that transcutaneous auricular vagus nerve stimulation, a form of electrical stimulation therapy, restored Lactobacillus abundance and improved symptoms in mice with constipation-predominant irritable bowel syndrome (Liu et al., 2024b). While these studies have focused on broad microbial composition, the specific strains involved in therapeutic modulation remain explored. Notably, our LEfSe analysis showed that EA treatment significantly increased the relative abundance of *Lactobacillus reuteri* in FC mice. *Lactobacillus reuteri* is a well-studied, internationally recognized probiotic strain widely used to treat various gastrointestinal diseases. Supplementation with *Lactobacillus reuteri* has been shown to increase bowel movement frequency, particularly in children with constipation (Kubota et al., 2020; Saviano et al., 2021). In recent decades, research on microbiota-gut motility interactions has predominantly relied on germ-free animal models. Given the specific nature of EA procedures, performing them in a completely sterile environment is challenging. Therefore, we used antibiotics to deplete gut bacteria as a low-cost, practical alternative to germ-free models. Consistent with a previous study (Xu et al., 2023), we found that broad-spectrum antibiotic impaired the beneficial effects of EA on defecation symptoms in constipated mice. Additionally, antibiotics inhibited the EA-induced modulation of *Lactobacillus reuteri* in the mouse intestine. These findings suggest that EA’s promotive effects on intestinal motility are dependent on the intestinal microbiota, particularly *Lactobacillus reuteri*. In addition, *Lactobacillus reuteri* treatment significantly increased the abundance of *Akkermansia muciniphila* and *Bifidobacterium animalis*. This phenomenon may highlight the pro-gastrodynamic role of *Lactobacillus reuteri* in regulating other intestinal flora. *Lactobacillus reuteri* directly regulates the intestinal microbiota by producing lactic acid and other metabolites and promotes the growth of beneficial flora by enhancing the intestinal barrier and modulating immune responses (He et al., 2019; Mu et al., 2018). For instance, *Akkermansia muciniphila* contributes to maintaining and regulating the intestinal mucosal barrier (Wang et al., 2020), while *Bifidobacterium* produces SCFAs via dietary fibers fermentation to support a healthy gut microenvironment (Zhang et al., 2024). The integrity of the intestinal mucosal barrier and microenvironment is the basis for normal intestinal function.

Recently, miRNAs have been identified in the feces of humans and animals, and their roles have been well characterized. Importantly, host-derived exosomes can transport miRNAs, enabling cross-species communication between prokaryotic and eukaryotic cells (Filip et al., 2016). Substantial evidence indicates that miRNAs regulate bacterial gene expression by destabilizing mRNA and inhibiting post-transcriptional protein synthesis, thereby influencing bacterial growth and metabolism (He et al., 2022; Liu et al., 2016). Our sequencing analysis revealed that miR-205-5p may promote the growth of *Lactobacillus reuteri* in constipated mice, which is regulated by EA. These findings suggest that miRNAs may act as critical mediators of acupuncture-specific modulation of the microbiome. miRNAs play a key role in host-microbe crosstalk (Dalmasso et al., 2011; Liu et al., 2016). Our findings show that even after gut microbiota depletion, EA continues to modulate miR-205-5p expression in fecal and colonic tissues, indicating its host-derived origin. Moreover, AAV9-mir205 intervention affected intestinal motility and microbial composition in normal mice and inhibited EA’s restorative effects on intestinal motility and *Lactobacillus reuteri* in constipated mice. Notably, *Lactobacillus reuteri* supplementation reversed reduction in intestinal motility caused by AAV9-miR205 intervention. These findings highlight the critical role of miR-205-5p in EA-mediated regulation of intestinal microbiota, particularly *Lactobacillus reuteri*, in restoring intestinal motility in constipation model mice.

*Lactobacillus reuteri* is a widely recognized and well-studied probiotic strain commonly used to treat gastrointestinal disorders, particularly those affecting motility (Kubota et al., 2020; Lai et al., 2024). Research indicates that *Lactobacillus reuteri* is involved in both the onset and progression of various diseases (Calvigioni et al., 2023; Montgomery et al., 2022). Thus, we focused on changes in the fecal metabolic profile of constipated mice with or without EA treatment. Analysis showed that the differential metabolites were predominantly involved in flavone and flavonol biosynthesis, tyrosine metabolism, tryptophan metabolism, and histidine metabolism. Among these, the tryptophan metabolic pathway is the most frequently reported regulatory pathway associated with *Lactobacillus reuteri* (Bender et al., 2023; Montgomery et al., 2022). This was further confirmed by the correlation analysis. This was further confirmed by the correlation analysis (Benech et al., 2021). Intestinal bacteria with tryptophanase activity metabolize tryptophan into indole or 5-hydroxyindole. Indole regulates the secretory function of colonic enteroendocrine L cells and activates enteroendocrine cells to produce 5-HT via the transient receptor potential ankyrin 1 (Trpa1) ion channel (Chimerel et al., 2014; Ye et al., 2021). 5-hydroxyindole regulates intestinal contractions through Ca^2+^ ion channels (Waclawiková et al., 2021). Bacteria with tryptophan decarboxylase activity metabolize tryptophan into tryptamine, which activates 5-HT receptors or promotes colonic anion-dependent fluid secretion, enhancing gastrointestinal motility (Bhattarai et al., 2018; Williams et al., 2014). Additionally, several tryptophan metabolites, including indole, indole lactic acid, and indole acetic acid, are identified as ligands of the aryl hydrocarbon receptor (AHR). The AHR signaling pathway has been implicated in regulating intestinal transit time in mice (Obata et al., 2020).

Targeted tryptophan metabolomics revealed that miR-205 knockdown via AAV9 altered EA’s regulation of tryptophan metabolites in FC mice, confirming that EA influences tryptophan metabolism through the miR-205 pathway. Moreover, AAV9-mediated miR-205 knockdown reduced fecal levels of indole-3-acetamide, indole-3-acetic acid, and indole-3-carboxaldehyde in normal mice, while *Lactobacillus reuteri* supplementation restored their levels. These findings suggest that *Lactobacillus reuteri* mediates the regulation of tryptophan metabolites (indole-3-acetamide, indole-3-acetic acid, and indole-3-carboxaldehyde) via host-derived miR-205-5p, a critical pathway for EA’s promotion of intestinal motility in FC mice. *Lactobacillus reuteri* metabolizes tryptophan, as shown by Montgomery et al., who found that adding tryptophan to an anaerobic culture of *Lactobacillus reuteri* resulted in the production of indole acetic salts (Montgomery et al., 2022). Additionally, *Lactobacillus reuteri* metabolizes tryptophan to produce indole-3-carboxaldehyde, which activates AHR in CD8^+^ T cells, exerting an immunomodulatory effect (Bender et al., 2023). Conversely, these tryptophan metabolites also regulate intestinal motility. These three metabolites activate AHR, which acts as a central hub linking gut environmental factors, such as microbiota, to enteric nervous system function (Wang L. et al., 2023; Wang M. et al., 2023; Wang et al., 2022; Zhang C. et al., 2023; Zhang X. et al., 2023). Research indicates that pharmacological or dietary regulation of AHR activity in enteric neurons can alleviate intestinal motility disorders (Obata et al., 2020). Furthermore, indole-3-carboxaldehyde activates the Trpa1 ion channel, which plays a role in regulating intestinal motility (Ye et al., 2021).

This study has several limitations. First, while this study primarily focused on the effects of miR-205-5p on gut function, miR-205-5p is also known to play a role in immune regulation, potentially influencing intestinal health indirectly. For instance, miR-205-5p regulates Kupffer cell M1 polarization by targeting retinoic acid receptor-related orphan receptor α in immune hepatic injury (Wang D. et al., 2024; Wang H. et al., 2024; Wang J. et al., 2024). Additionally, miR-205-5p is implicated in epithelial-mesenchymal transition in the lungs by competitively binding to circ_000999 (Wang D. et al., 2024; Wang H. et al., 2024; Wang J. et al., 2024). Therefore, whether targeted intervention of intestinal miR-205-5p or its role in improving constipation through immune response modulation requires further validation. Second, the study design did not include a separate Abx group or FC + Abx group to specifically examine the effects of antibiotics on the constipated mouse model. Although we compared the FC + EA and FC + EA + Abx groups to explore the effect of antibiotics on EA treatment for constipation, the absence of an Abx or FC + Abx group limited our ability to fully evaluate the independent effects of antibiotics on gut microbiota and their interaction with EA treatment in improving constipation symptoms. Future studies should include these two groups to systematically evaluate the independent effects of antibiotics on gut microbiota and treatment outcomes. Third, while this study focused on miR-205-5p, the roles of other miRNAs in gut microbiota and FC remain of significant interest. Future research should investigate the potential roles of other miRNAs, particularly their synergistic effects in various pathological contexts, to develop comprehensive targeted therapeutic strategies.

## 5 Conclusion

**Figure 7:**
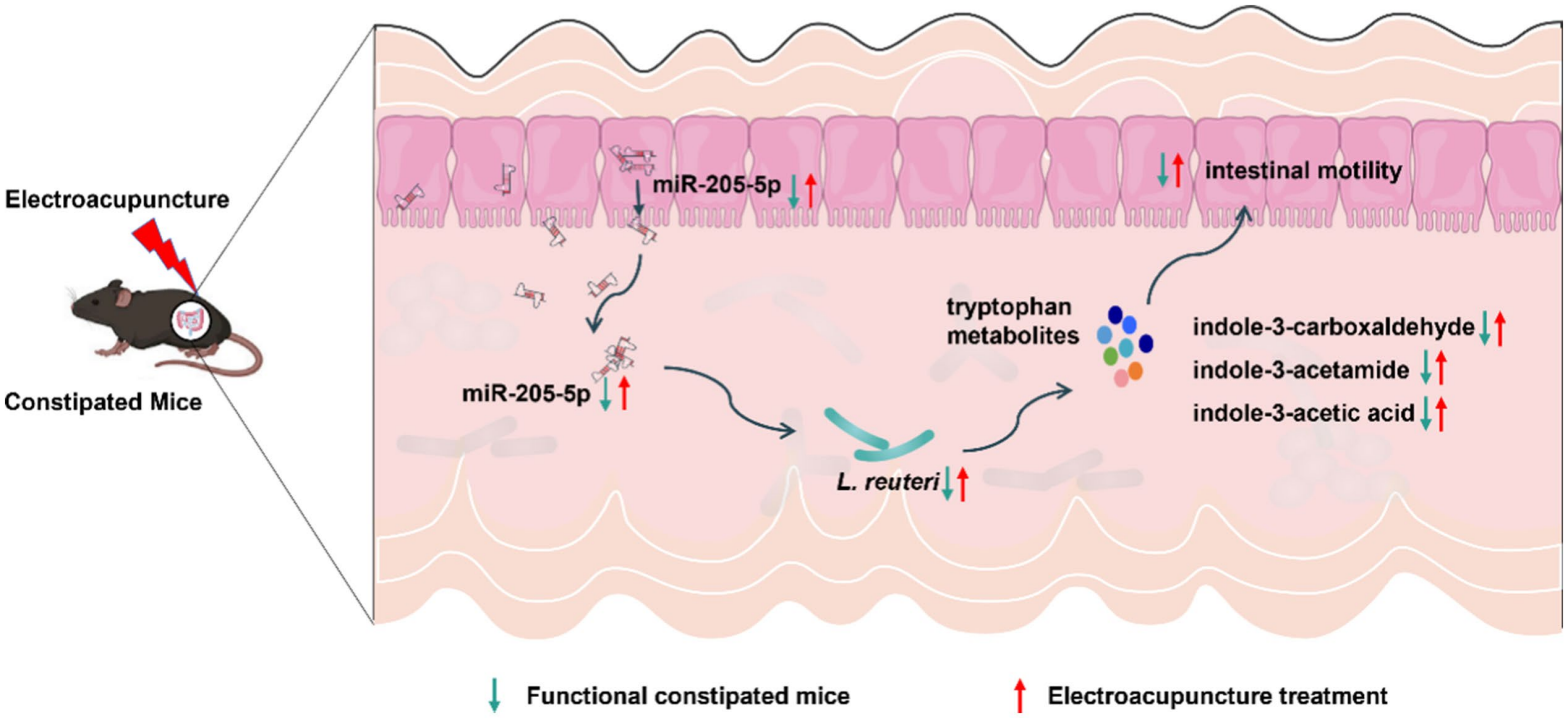
Potential mechanisms of EA in the treatment of FC.

In summary, using multi-omics screening and validation, we demonstrated that EA upregulates host-derived miR-205-5p, promotes the growth of *Lactobacillus reuteri*, and modulates tryptophan metabolites—specifically indole-3-acetamide, indole-3-acetic acid, and indole-3-carboxaldehyde—to enhance gut motility in a loperamide-induced constipation mouse model (Figure 7). This study not only offers new insights into the mechanisms underlying EA’s efficacy in constipation treatment but also suggests a novel therapeutic strategy combining EA with probiotics for disease prevention and management.

## Data Availability

The original contributions presented in the study are publicly available. This data can be found here: https://ngdc.cncb.ac.cn/, accession number PRJCA034916.
