# Supplementary material for: Electroacupuncture alleviates functional constipation by upregulating host-derived miR-205-5p to modulate gut microbiota and tryptophan metabolism
Source: Front Microbiol. 2025 Feb 5;16:1517018. doi: 10.3389/fmicb.2025.1517018 (PMC11835812; doi:10.3389/fmicb.2025.1517018)

**Supplemental Information**

**Supplemental Table 1**: Primers for quantification of miRNAs expression and bacterial gene transcript by qPCR

| Gene | 5’-3’ Primer sequence |
| --- | --- |
| miR-205-5p | F1-TCCTTCATTCCACCGGAGTCT |
|  | UR-PloyA -GCTGTCAACGATACGCTACGTAAC |
| U6 | F-TCGCTTCGGCAGCACATATAC |
|  | R-ATATGGAACGCTTCACGAATTTG |
| *Lactobacillus reuteri* 16S | F- GTGTAGCGGTGGAATGCGTAG |
|  | R- CGAGCCTCAGCGTCAGTTG |

**Supplemental Table 2**: Differentially expressed miRNAs compared between NC group and FC group

|  | **miRNA ID** | **Log2FoldChange** | **adjust *P* value** |
| --- | --- | --- | --- |
| **Down** | mmu-miR-490-3p | -1.522360643 | 1.13E-06 |
|  | mmu-miR-490-5p | -1.404126573 | 0.001562277 |
|  | mmu-miR-148a-3p | -1.111385383 | 0.00047366 |
|  | mmu-miR-148a-5p | -0.820225586 | 0.047958806 |
| **Up** | mmu-miR-615-3p | 4.077708257 | 2.93E-05 |
|  | mmu-miR-10a-3p | 2.878069489 | 3.75E-21 |
|  | mmu-miR-10b-3p | 2.780296318 | 1.06E-10 |
|  | mmu-miR-10b-5p | 2.704587375 | 5.35E-10 |
|  | mmu-miR-10a-5p | 2.558803872 | 3.95E-13 |
|  | mmu-miR-672-5p | 1.793771493 | 0.005964576 |
|  | mmu-miR-326-3p | 1.654323885 | 3.43E-05 |
|  | mmu-miR-205-5p | 1.219581023 | 0.005964576 |
|  | mmu-miR-224-5p | 1.129202269 | 0.031116045 |
|  | mmu-miR-708-5p | 0.71979714 | 0.031116045 |
|  | mmu-miR-7b-5p | 0.637261106 | 0.031116045 |

**Supplemental Table 3**: Differentially expressed miRNAs compared between FC+EA group and FC group

|  | **miRNA ID** | **Log2FoldChange** | **adjust *P* value** |
| --- | --- | --- | --- |
| **Down** | mmu-miR-490-5p | -1.930845296 | 0.000432854 |
|  | mmu-miR-490-3p | -1.373478116 | 3.53E-07 |
|  | mmu-miR-190a-5p | -1.26345529 | 0.016149957 |
|  | mmu-miR-494-3p | -1.169361579 | 0.046326162 |
|  | mmu-miR-376b-3p | -1.030446292 | 0.046326162 |
|  | mmu-miR-148a-5p | -1.009292733 | 0.005314737 |
|  | mmu-miR-96-5p | -0.877898083 | 0.03360371 |
|  | mmu-miR-299a-3p | -0.858802778 | 0.011524869 |
|  | mmu-miR-183-5p | -0.710116675 | 0.026892807 |
| **Up** | mmu-miR-615-3p | 4.700323422 | 7.51E-08 |
|  | mmu-miR-10b-3p | 3.460329394 | 3.62E-13 |
|  | mmu-miR-10a-3p | 3.042497283 | 7.31E-20 |
|  | mmu-miR-450b-3p | 2.941879169 | 0.008286225 |
|  | mmu-miR-672-5p | 2.459817718 | 9.70E-07 |
|  | mmu-miR-10b-5p | 2.37853308 | 2.56E-09 |
|  | mmu-miR-690 | 2.152069461 | 0.007490416 |
|  | mmu-miR-10a-5p | 2.145813583 | 2.11E-08 |
|  | mmu-miR-224-5p | 1.834614678 | 7.46E-07 |
|  | mmu-miR-3473a | 1.694322214 | 0.017663875 |
|  | mmu-miR-205-5p | 1.484783901 | 0.003164008 |
|  | mmu-miR-708-5p | 1.460799642 | 0.000904309 |
|  | mmu-miR-128-3p | 1.112695756 | 0.006702244 |
|  | mmu-miR-421-3p | 1.058037076 | 0.038142676 |

**Supplemental Table 4**: Comparison of tryptophan metabolites between AAV9-ctrl+FC+EA group and AAV9-mir205+FC+EA group

| **NO.** | **Tryptophan metabolites** | **AAV9-ctrl+FC**  **+EA group（8）** | **AAV9-mir205+FC**  **+EA group（7）** |
| --- | --- | --- | --- |
| 1 | Picolinic acid (PCL 016) | 0.299±0.105 | 0.205±0.028^*^ |
| 2 | 5-Hydroxyanthranilic Acid | 0.011±0.002 | 0.011±0.002 |
| 3 | Nicotinic Acid | 10.681±2.117 | 10.971±2.760 |
| 4 | Quinolinic acid | 0.046±0.032 | 0.025±0.019 |
| 5 | 2-Aminophenol | 0.031±0.007 | 0.029±0.004 |
| 6 | Nicotinamide (Vitamin B3) | 0.188±0.034 | 0.203±0.069 |
| 7 | 3-Hydroxy-DL-kynurenine | 0.017±0.005 | 0.018±0.003 |
| 8 | 5-hydroxytryptophan | 0.034±0.008 | 0.025±0.008 |
| 9 | Serotonin | 0.118±0.091 | 0.078±0.025 |
| 10 | rac-Kynurenine | 0.090±0.024 | 0.054±0.021^*^ |
| 11 | 3-hydroxyanthranilic acid | 0.004±0.001 | 0.004±0.001 |
| 12 | Xanthurenic acid | 0.424±0.339 | 0.212±0.026 |
| 13 | L-Tryptophan | 20.584±10.354 | 13.063±8.469 |
| 14 | Kynurenic Acid | 0.470±0.143 | 0.285±0.071^**^ |
| 15 | Tryptamine | 0.122±0.169 | 0.518±0.393^*^ |
| 16 | 5-Hydroxyindole-3-aceticAcid | 0.165±0.072 | 0.125±0.044 |
| 17 | N-Acetyl-serotonin | 0.008±0.005 | 0.005±0.001 |
| 18 | Indole-3-acetamide | 0.008±0.003 | 0.004±0.001^**^ |
| 19 | Indole-3-lactic Acid | 1.041±1.029 | 0.632±0.688 |
| 20 | N-(3-Indolylacetyl)-L-alanine | 0.0011±0.0005 | 0.0004±0.0003^*^ |
| 21 | Indole-3-carboxylic acid | 0.078±0.036 | 0.054±0.008 |
| 22 | Indole-3-carboxaldehyde | 1.97±0.683 | 0.951±0.302^**^ |
| 23 | Indole-3-acetic acid | 1.988±0.515 | 1.200±0.352^**^ |
| 24 | Tryptophol | 0.073±0.052 | 0.051±0.021 |
| 25 | Cinnabarinic Acid | 0.005±0.001 | 0.005±0.001 |
| 26 | 3-Indoleacrylicacid | 0.013±0.014 | 0.020±0.014 |
| 27 | 3-Indolepropionic acid | 0.290±0.127 | 0.261±0.097 |
| 28 | 3-Indoleacetonitrile | 0.002±0.001 | 0.001±0.002 |
| 29 | Indole | 1.485±0.866 | 1.071±0.786 |
| 30 | 3-Methylindole | 0.413±0.270 | 0.352±0.236 |
| 31 | Indoxyl Sulfate Potassium Salt | 0.327±0.803 | 0.058±0.126 |

Note：vs AAV9-ctrl +FC+EA group: ^*^*P*<0.05，^**^*P*<0.01.

**Supplemental Table 5**: Comparison of tryptophan metabolites between AAV9-ctrl group and AAV9-mir205 group

| **NO.** | **Tryptophan metabolites** | **AAV9-ctrl group (8)** | **AAV9-mir205 group (8)** |
| --- | --- | --- | --- |
| 1 | Picolinic acid (PCL 016) | 0.373±0.090 | 0.321±0.128 |
| 2 | 5-Hydroxyanthranilic Acid | 0.015±0.005 | 0.018±0.009 |
| 3 | Nicotinic Acid | 12.11±2.23 | 10.43±1.69 |
| 4 | Quinolinic acid | 0.109±0.049 | 0.102±0.064 |
| 5 | 2-Aminophenol | 0.042±0.016 | 0.059±0.022 |
| 6 | Nicotinamide (Vitamin B3) | 0.231±0.062 | 0.196±0.056 |
| 7 | 3-Hydroxy-DL-kynurenine | 0.017±0.005 | 0.019±0.005 |
| 8 | 5-hydroxytryptophan | 0.064±0.038 | 0.065±0.041 |
| 9 | Serotonin | 0.064±0.031 | 0.116±0.096 |
| 10 | rac-Kynurenine | 0.086±0.043 | 0.097±0.034 |
| 11 | 3-hydroxyanthranilic acid | 0.007±0.004 | 0.007±0.006 |
| 12 | Xanthurenic acid | 0.520±0.267 | 0.562±0.193 |
| 13 | L-Tryptophan | 12.93±5.47 | 10.20±4.93 |
| 14 | Kynurenic Acid | 0.541±0.230 | 0.601±0.208 |
| 15 | Tryptamine | 0.264±0.489 | 0.280±0.484 |
| 16 | 5-Hydroxyindole-3-aceticAcid | 0.223±0.175 | 0.295±0.164 |
| 17 | N-Acetyl-serotonin | 0.007±0.004 | 0.009±0.006 |
| 18 | Indole-3-acetamide | 0.008±0.004 | 0.005±0.002^*^ |
| 19 | Indole-3-lactic Acid | 1.114±1.700 | 0.736±0.830 |
| 20 | N-(3-Indolylacetyl)-L-alanine | 0.001±0.001 | 0.001±0.001 |
| 21 | Indole-3-carboxylic acid | 0.104±0.034 | 0.066±0.025^*^ |
| 22 | Indole-3-carboxaldehyde | 1.525±0.691 | 0.768±0.356^*^ |
| 23 | Indole-3-acetic acid | 2.884±1.328 | 1.609±0.720^*^ |
| 24 | Tryptophol | 0.089±0.029 | 0.056±0.017^*^ |
| 25 | Cinnabarinic Acid | 0.005±0.001 | 0.006±0.001 |
| 26 | 3-Indoleacrylicacid | 0.041±0.031 | 0.016±0.015 |
| 27 | 3-Indolepropionic acid | 0.774±0.524 | 0.364±0.141 |
| 28 | 3-Indoleacetonitrile | 0.001±0.001 | 0.001±0.001 |
| 29 | Indole | 0.517±0.392 | 1.344±0.799^*^ |
| 30 | 3-Methylindole | 0.229±0.099 | 0.214±0.160 |
| 31 | Indoxyl Sulfate Potassium Salt | 0.023±0.013 | 0.036±0.033 |

Note: vs AAV9-ctrl group: ^*^P<0.05.

**Supplemental Table 6**: Comparison of tryptophan metabolites between AAV9-mir205 group and AAV9-mir205+LR group

| **NO.** | **Tryptophan metabolites** | **AAV9-mir205 group**  **(6)** | **AAV9-mir205+LR group (6)** |
| --- | --- | --- | --- |
| 1 | Picolinic acid (PCL 016) | 0.300±0.117 | 0.324±0.117 |
| 2 | 5-Hydroxyanthranilic Acid | 0.019±0.009 | 0.011±0.003 |
| 3 | Nicotinic Acid | 10.52±1.92 | 11.56±3.95 |
| 4 | Quinolinic acid | 0.112±0.072 | 0.034±0.015 |
| 5 | 2-Aminophenol | 0.069±0.016 | 0.031±0.009^***^ |
| 6 | Nicotinamide (Vitamin B3) | 0.205±0.060 | 0.263±0.172 |
| 7 | 3-Hydroxy-DL-kynurenine | 0.018±0.004 | 0.015±0.002 |
| 8 | 5-hydroxytryptophan | 0.068±0.046 | 0.024±0.008 |
| 9 | Serotonin | 0.138±0.102 | 0.073±0.027 |
| 10 | rac-Kynurenine | 0.095±0.036 | 0.048±0.0135^*^ |
| 11 | 3-hydroxyanthranilic acid | 0.009±0.007 | 0.004±0.002 |
| 12 | Xanthurenic acid | 0.590±0.170 | 0.225±0.107^**^ |
| 13 | L-Tryptophan | 8.283±1.526 | 10.955±1.530^*^ |
| 14 | Kynurenic Acid | 0.555±0.225 | 0.282±0.097^*^ |
| 15 | Tryptamine | 0.369±0.539 | 0.469±0.407 |
| 16 | 5-Hydroxyindole-3-aceticAcid | 0.359±0.134 | 0.171±0.070^*^ |
| 17 | N-Acetyl-serotonin | 0.011±0.006 | 0.010±0.002 |
| 18 | Indole-3-acetamide | 0.005±0.002 | 0.010±0.004^*^ |
| 19 | Indole-3-lactic Acid | 0.483±0.302 | 0.683±1.533 |
| 20 | N-(3-Indolylacetyl)-L-alanine | 0.0006±0.0003 | 0.0005±0.0002 |
| 21 | Indole-3-carboxylic acid | 0.058±0.017 | 0.090±0.033 |
| 22 | Indole-3-carboxaldehyde | 0.637±0.079 | 1.588±0.453^**^ |
| 23 | Indole-3-acetic acid | 1.467±0.755 | 2.223±0.695^*^ |
| 24 | Tryptophol | 0.051±0.013 | 0.068±0.043 |
| 25 | Cinnabarinic Acid | 0.006±0.001 | 0.005±0.001 |
| 26 | 3-Indoleacrylicacid | 0.013±0.011 | 0.015±0.009 |
| 27 | 3-Indolepropionic acid | 0.330±0.150 | 0.480±0.308 |
| 28 | 3-Indoleacetonitrile | 0.002±0.001 | 0.001±0.001 |
| 29 | Indole | 1.158±0.427 | 1.074±1.090 |
| 30 | 3-Methylindole | 0.162±0.071 | 0.201±0.103 |
| 31 | Indoxyl Sulfate Potassium Salt | 0.031±0.026 | 0.005±0.002 |

Note: vs AAV9-mir205 group: ^*^*P*<0.05，^**^*P*<0.01，^***^*P*<0.001.

**Supplemental Figure 1:** Comparisons of excretion parameters before and after EA treatment


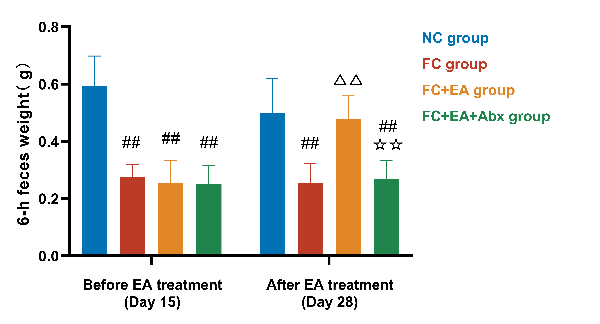

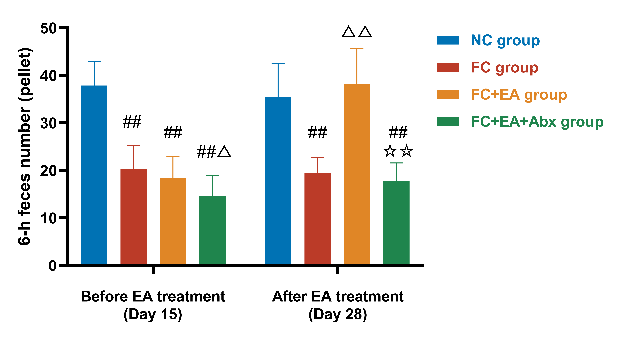


Data are expressed as mean ± standard deviation, n = 8. vs NC group：^##^*P*＜0.01；vs FC group：^△△^*P*＜0.01；vs FC+EA group：^☆☆^*P*＜0.01. EA: electroacupuncture; FC: functional constipation; NC: normal control; Abx: antibiotic cocktail.

**Supplemental Figure 2:** Comparisons of alpha diversity between groups


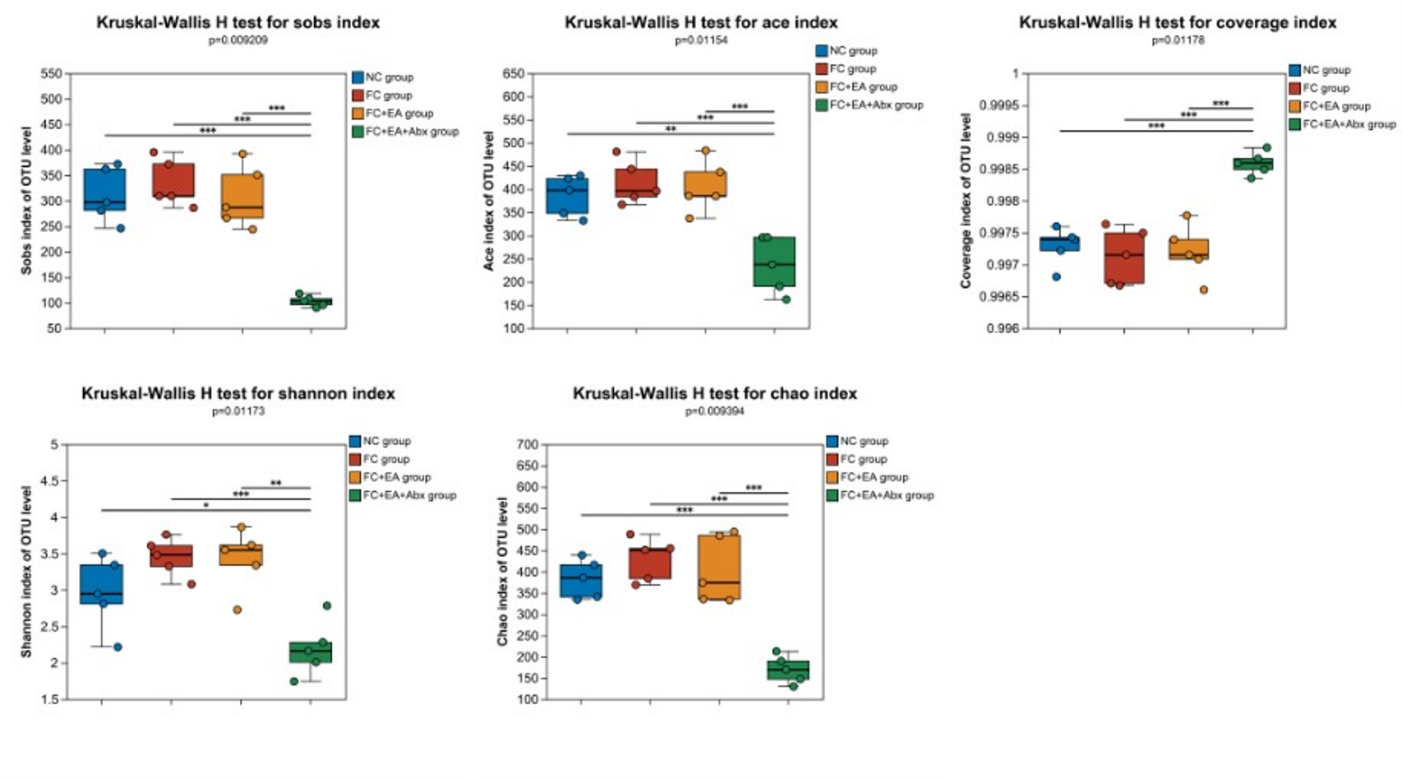
Data are expressed as mean ± standard deviation, n = 8. ^*^*P*＜0.05; ^**^*P*＜0.01; ^***^*P*＜0.001. EA: electroacupuncture; FC: functional constipation; NC: normal control; Abx: antibiotic cocktail.

**Supplemental Figure 3:** Linear discriminant analysis Effect Size (LEfSe) analysis between groups


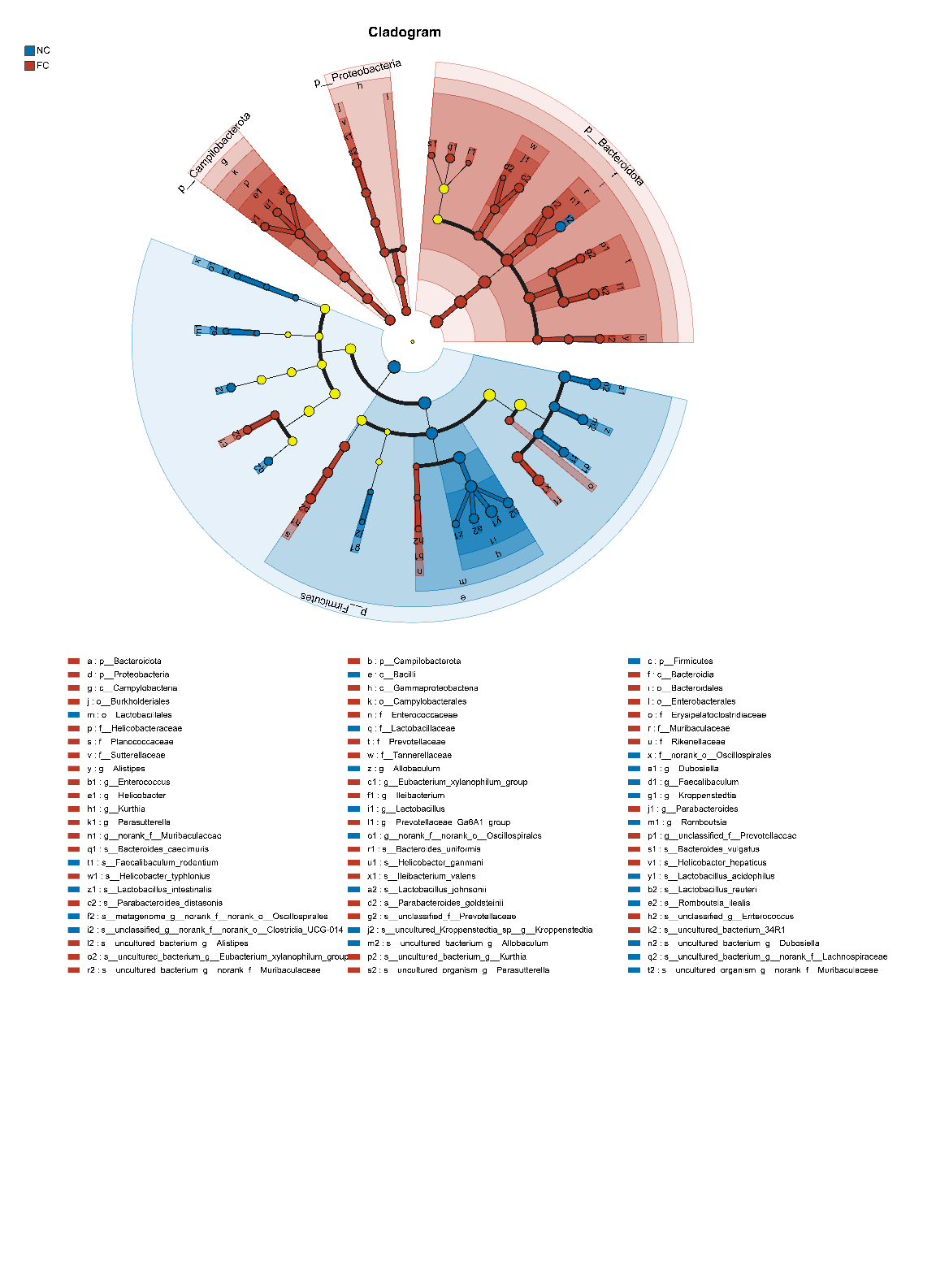


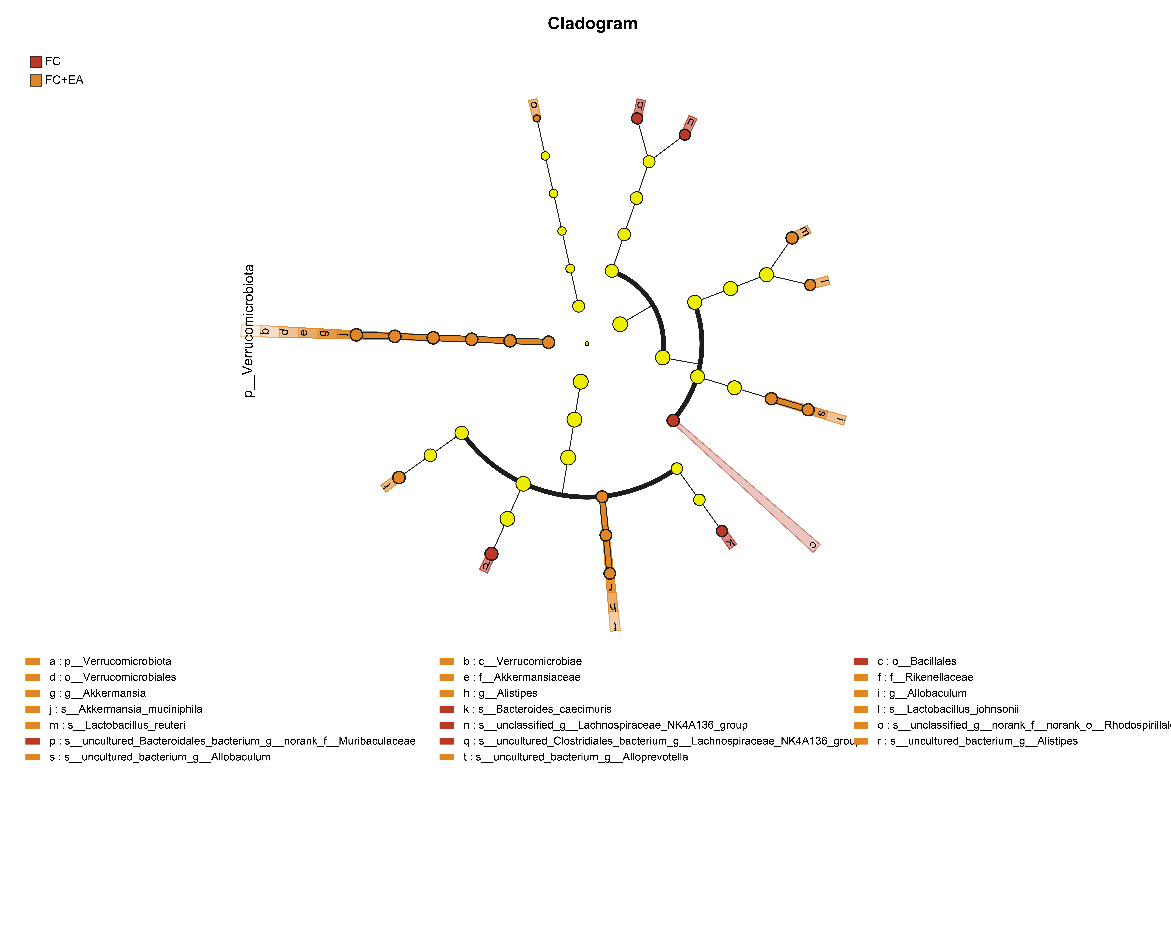


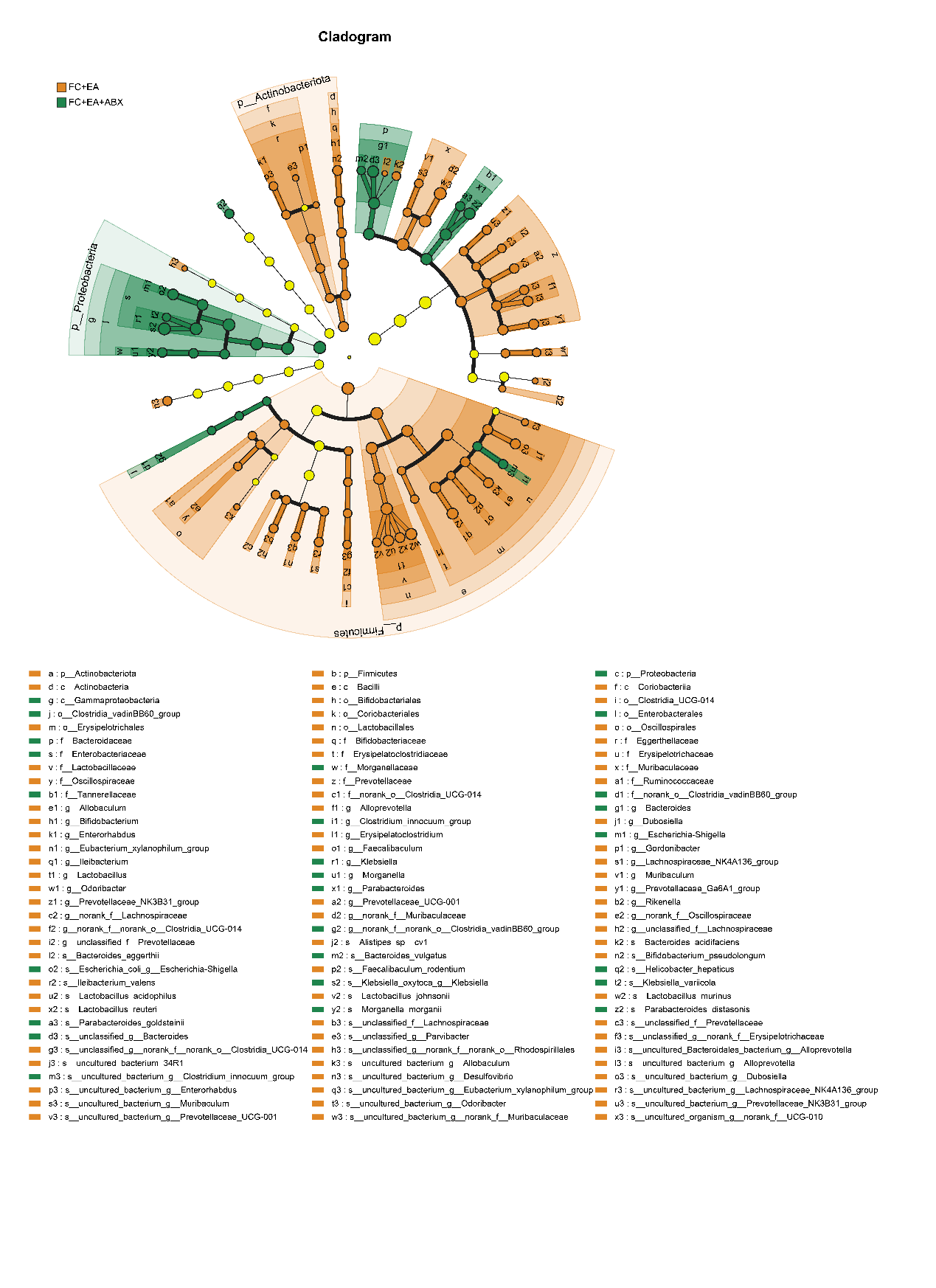


**Supplemental Figure 4:** Comparisons of alpha diversity between AAV9-ctrl+FC+EA group and AAV9-mir205+FC+EA group


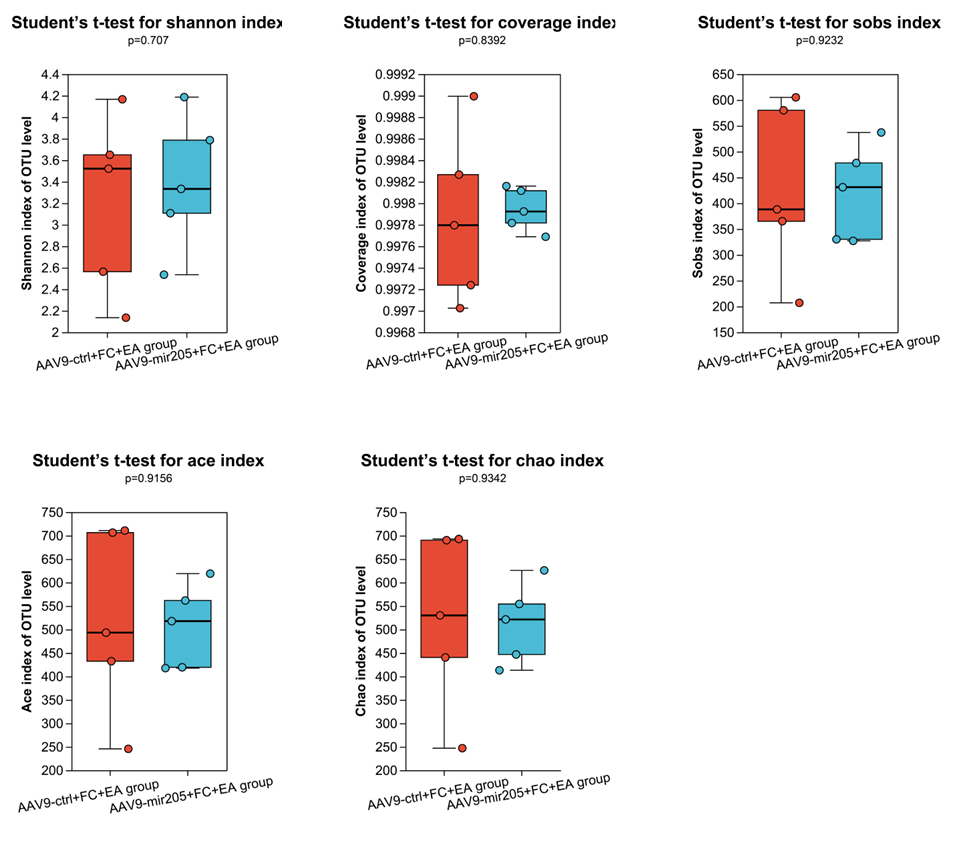


**Supplemental Figure 5:** Comparisons of alpha diversity in AAV9-ctrl group, AAV9-mir205 group and AAV9-mir205+LR group


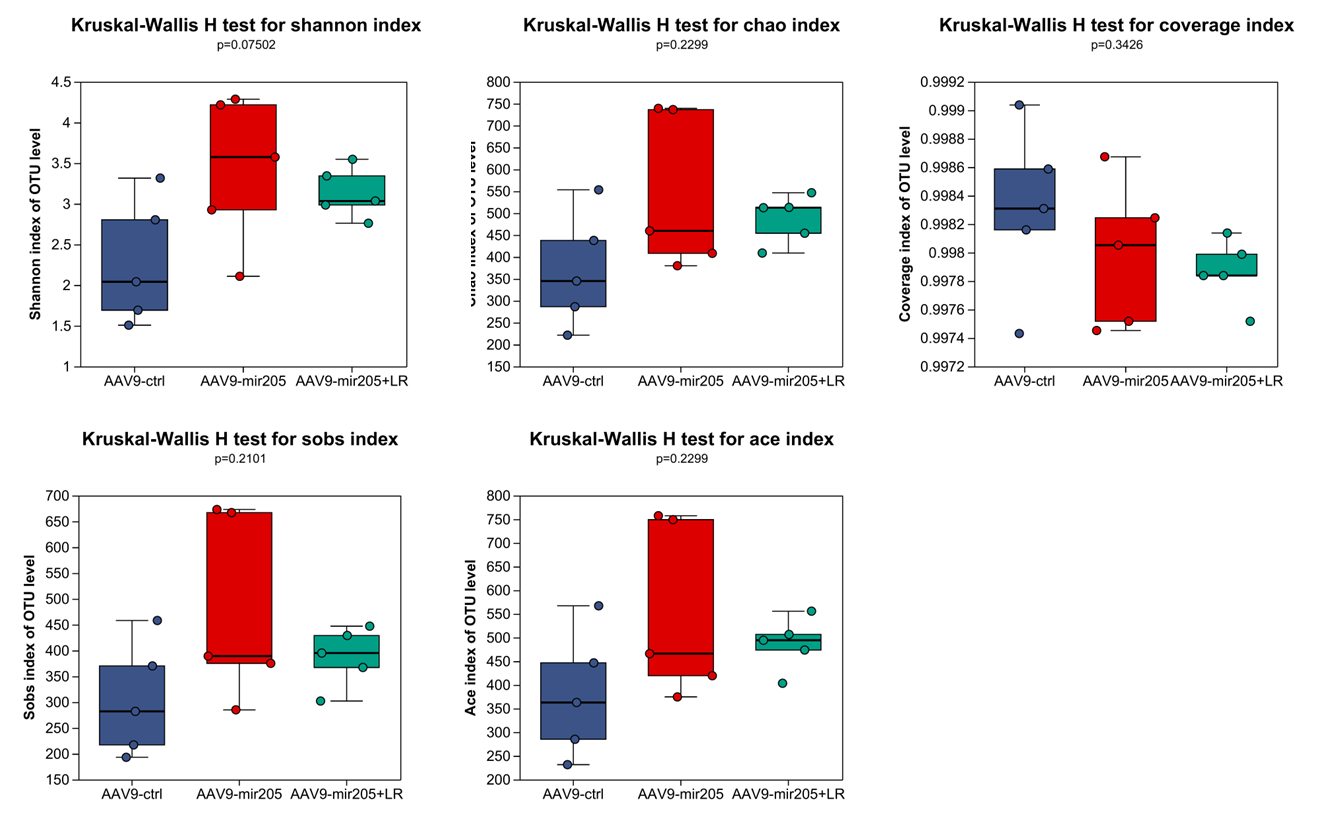


**Supplemental Figure 6:** Linear discriminant analysis Effect Size (LEfSe) analysis between AAV9-ctrl group and AAV9-mir205 group


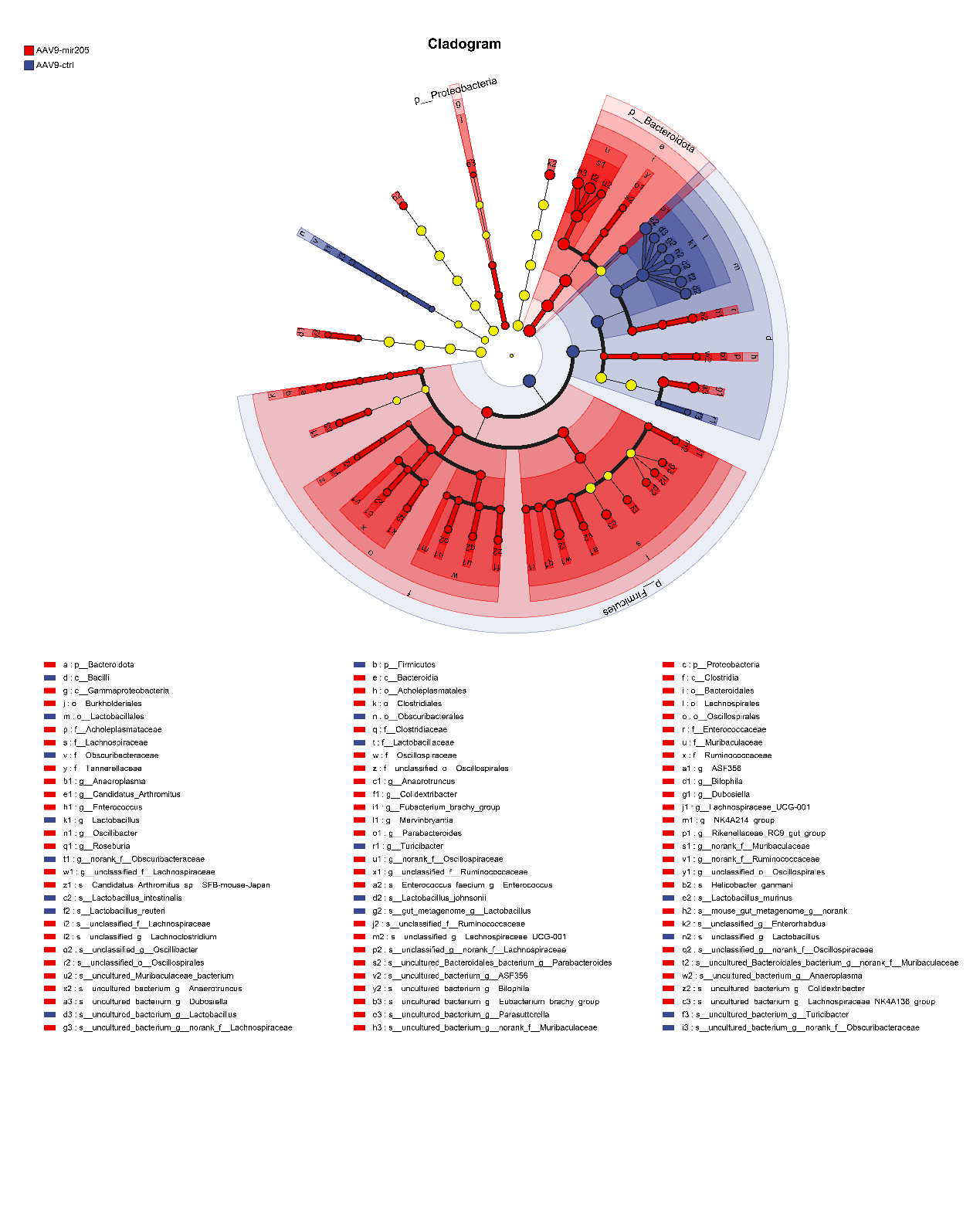


**Supplemental Figure 7:** Linear discriminant analysis Effect Size (LEfSe) analysis between AAV9-mir205 group and AAV9-mir205+LR group


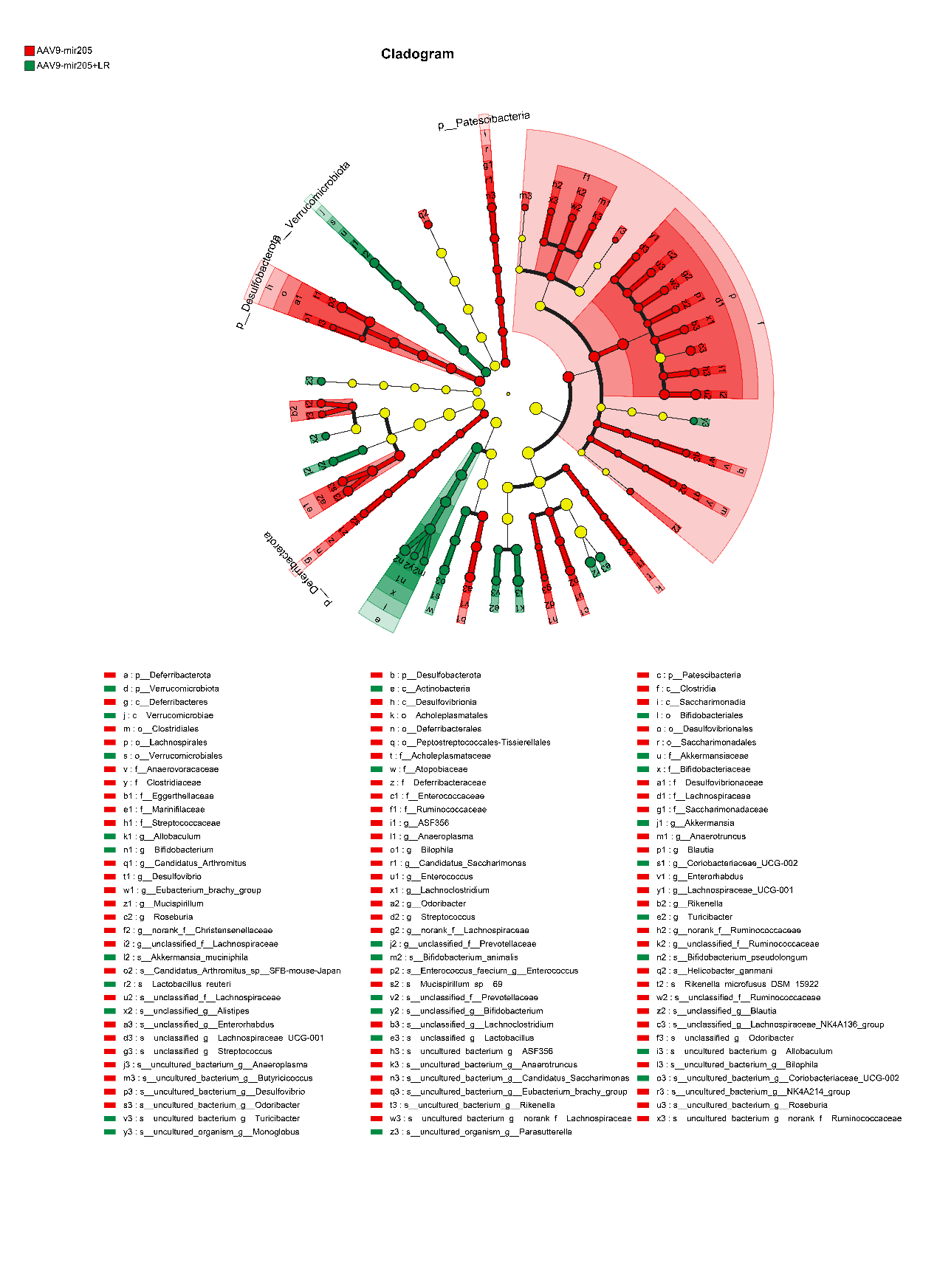


**Supplemental Figure 8:** Metabolic profile analysis between groups by OPLS-DA analysis


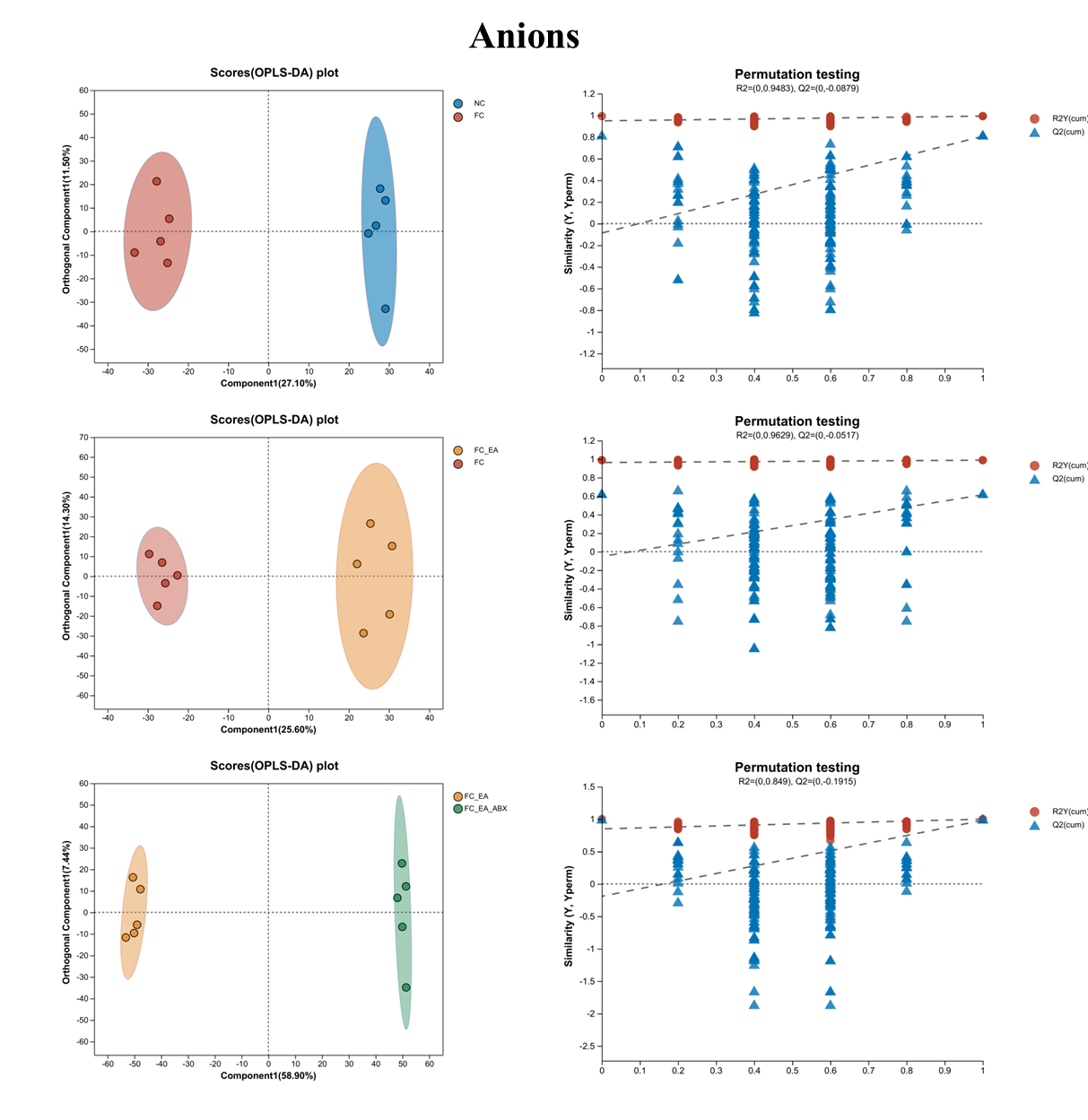


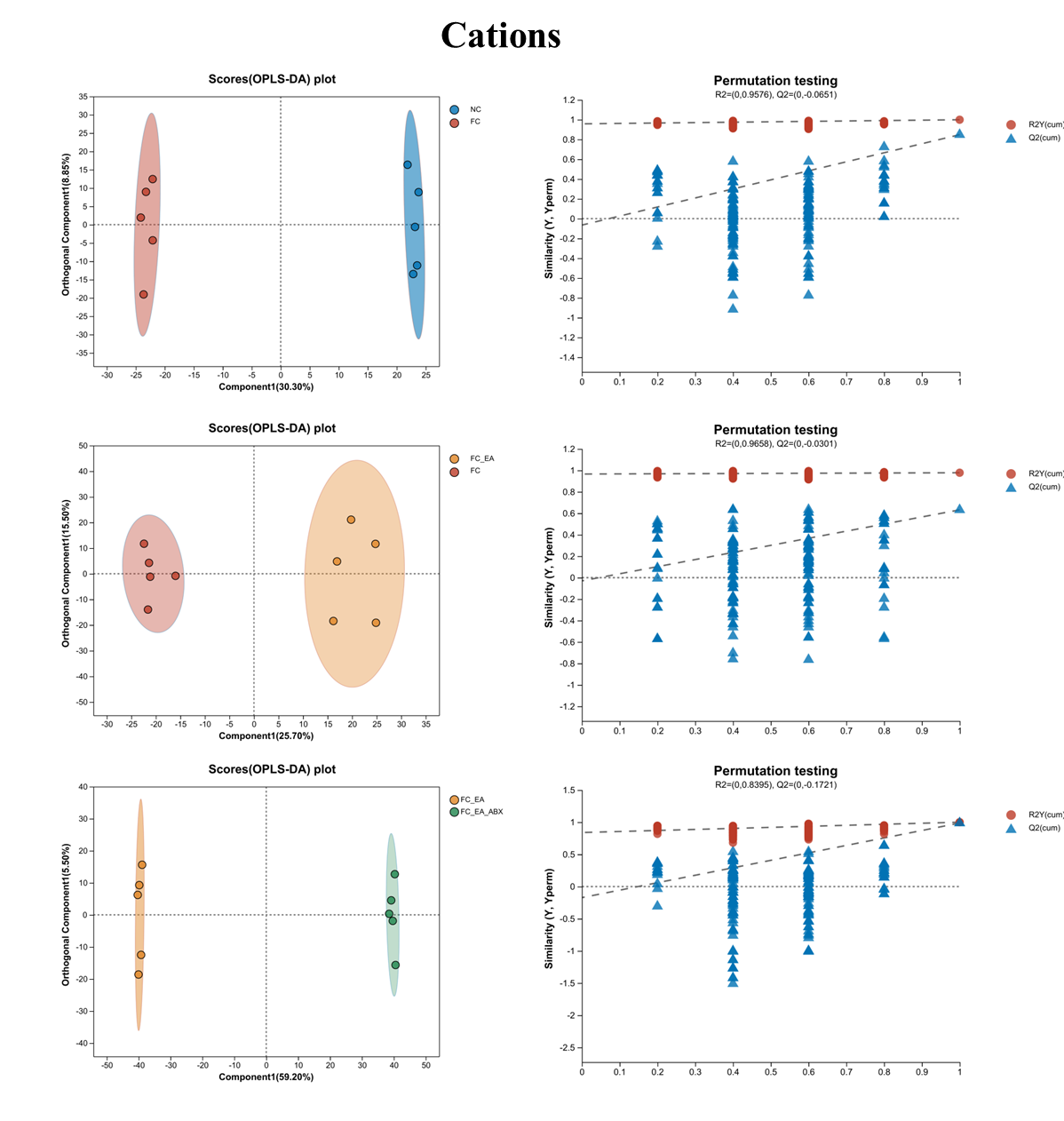

Supplement: Supplementary file 1 [file Supplementary_file_1.docx]
